# MCMCINLA estimation of varying coefficient spatial lag model—A study of China’s economic development in the context of population aging

**DOI:** 10.1371/journal.pone.0279504

**Published:** 2023-05-15

**Authors:** Jiaqi Teng, Shuzhen Ding, Huiguo Zhang, Xijian Hu

**Affiliations:** College of Mathematics and System Science, Xinjiang University, Urumqi, Xinjiang, China; National Taiwan University, TAIWAN

## Abstract

The dominant spatial econometric model in spatial econometrics is the parametric form, while in the realistic context, the variables often do not satisfy the assumption of linearity and have nonlinear relationships with each other. In this paper, we introduce nonparametric terms into spatial econometric models and propose the MCMCINLA estimation method for varying coefficient spatial lag models. The empirical analysis is conducted with the socioeconomic data of mainland China from 2015 to 2020 to discuss the influencing factors and spatial and temporal distribution characteristics of China’s economic development under the classical spatial lag model and the varying coefficient spatial lag model with population aging as a special covariate, respectively. The results show that with the gradual aging of the population, foreign trade will inhibit the development of regional economy to a certain extent, while urbanization process, resident income, real estate development and high-tech development will have a driving effect on economic growth, and high-tech development has the strongest mobilization on regional economic development. Compared with the classical spatial lag model, the varying coefficient spatial lag model can more fully exploit the information of variables in a more realistic context and derive the variable evolution process.

## 1. Introduction

In many fields such as ecology, epidemiology and economics, the observations obtained often have certain geospatial characteristics, and the models established often have both spatial correlation and spatial heterogeneity, when the traditional econometric methods are no longer applicable because they cannot distinguish between spatial correlation and spatial heterogeneity, and spatial econometrics comes into being. In recent years, the spatial element has received increasing attention in the empirical evidence of economics, and spatial econometric models have become a standard analytical tool for spatial data and have been widely used in regional economics [1], ecology [2], and sociology [3].

The classical spatial econometric models mainly make linear assumptions and model spatial correlation or spatial spillover effects, while in the real context there is not necessarily a linear relationship between variables, but more often nonlinear characteristics [4]. Therefore, it is necessary to introduce nonparametric terms into the spatial econometric models.

The spatial varying coefficient model is a generalization of the regression model, which was first given by Brunsdon et al. (1996) [5], and proposed a nonparametric smooth estimation method with the distance function between the observation points as the weight—Geographically weighted regression (GWR) method. He introduced the spatial properties of the data into the regression coefficients, assuming that the regression coefficients are a function of the spatial coordinates of each observation point, and explored and analyzed the spatial non-stationarity of the regression relationship by estimating the value of the coefficient function at each position. Fotheringham et al. (2002) [6] systematically summarized the principle and statistical inference of GWR method for the first time, and discussed the application of GWR in generalized linear models. In addition, the development of GWR further expands the related research on nonparametric spatial econometric models. For instance, Cho et al. (2010) [7] proposed a GWR-SLM varying coefficient model that combines GWR with spatial lag model; Basile et al. (2014) [8] considered the existence of spatial correlation, spatial heterogeneity and nonlinearity simultaneously, and obtained a new penalized spline spatial lag geo-additive model (PS-SLM); Geniaux et al. (2018) [9] presented a new hybrid spatial geo-weighted lag model (M-GWR-SLM) which allows regression parameters and spatial dependencies to vary over space. With the gradual development of spatial statistics and Bayesian statistics, the spatially varying coefficient Bayesian hierarchical model has become an effective alternative to GWR and has been favored. Gelfand et al. (2003) [10] focused around spatially varying coefficient Bayesian hierarchical models and provided extensions to generalized linear models and spatial-temporal settings; Bakar et al. (2016) [11] developed an R package, spTDyn, for modeling spatially varying coefficients and temporal dynamic processes in Bayesian hierarchical models; Kroc (2017) [12] explored the habitat behavior characteristics of moose with the help of a spatially varying coefficient Bayesian hierarchical model.

Due to the existence of spatial coordinates, the models discussed above can easily obtain the variation characteristics of the response variable with the spatial location, but it is not easy to obtain the dynamic variation law of the response variable with a covariate. In order to deeply explore the regression relationship between variables, this paper focuses on such models in which the coefficient function is an unknown function of a covariate, that is, by constructing a varying coefficient spatial lag model for research and analysis. The model has the advantages of high flexibility and strong adaptability, it can effectively avoid the problem of the curse of dimensionality, and can obtain the dynamic change characteristics of the response variable and other covariates under the time lapse of a covariate, which is especially suitable for use in longitudinal data analysis.

Spatial lag model is the most widely used type of spatial econometric model. It is used to embody spatial autocorrelation and can reflect the impact of neighboring regions on the region. Currently, estimation on nonparametric spatial lag models is flourishing, and various estimation methods have been proposed successively. Su and Jin (2010) [13] estimated the parametric and nonparametric components of a semiparametric spatial lag model using Local Linear and Cross-Sectional Likelihood methods, respectively. Li and Chen (2013) [14] proposed a new class of semiparametric varying coefficient spatial lag models by setting the coefficients of the explanatory variables in the form of unknown functions of certain variables and estimated the model parameters with the help of Contour Maximum Likelihood. Fang and Qian (2013) [15] estimated the nonparametric spatial lag model by using inverse jump MCMC. Chen et al. (2015, 2016) [16, 17] successively proposed Two-Stage Least Squares estimation and General Moment estimation for semiparametric panel spatial lag models. Krisztin (2017) [18] combined penalized splines with Bayesian methods to propose a new Bayesian semiparametric estimation method for semiparametric spatial lag models. Li and Chen (2018) [19] constructed a nonparametric spatial lag model with random independent variables and gave a General Moment estimation for this model. Zhao and Pu (2021) [20] proposed a new spatial lag single indicator varying coefficient model and completed the nonparametric estimation process by combining spline and Maximum Likelihood.

Bayesian estimation plays an increasingly important role in the statistical inference of spatial econometric models, as the next major parameter estimation method after Maximum Likelihood estimation and Moment estimation. Compared with Maximum Likelihood estimation and Moment estimation, Bayesian estimation is more adaptable to data. Maximum Likelihood estimation finds parameters by maximizing the likelihood function, which is more dependent on the data. When the amount of data is large, the Maximum Likelihood can estimate the parameters well; while when the amount of data is small, the Maximum Likelihood estimation result may not be ideal. Bayesian estimation uses probability to describe the change of unknown parameters. It regards the parameter as a random variable. By introducing a priori to estimate the parameter, a good estimation effect can still be obtained in the case of a small amount of data [21].

For Bayesian statistics, although it can better complete the parameter estimation in the case of small amount of data, it also has the problem of complex calculation process, especially the inference and calculation of the marginal posterior distribution of unknown parameters has always been a major difficulty in Bayesian statistics. In the early 1990s, Robert et al. [22] made a major breakthrough in Bayesian inference by applying the Markov Chain Monte Carlo (MCMC) method through inference simulation. MCMC can solve sampling difficulties such as high-dimensional integration, but may be limited by convergence speed and numerical stability when facing large models or large amounts of data. To address this problem, Rue et al. (2009) [23] proposed Integrated Nested Laplace Approximation (INLA), an algorithm that combines Laplace approximation with modern numerical integration in a Bayesian framework, which can greatly shorten the computation time and improve the computation efficiency and reduce the computation cost while guaranteeing the estimation accuracy of MCMC algorithm. Bivand et al. (2014) [24] extended INLA by Bayesian Model Averaging (BMA) for spatial econometric models and developed an INLABMA package. Gómez-Rubio et al. (2017) [25] described the implementation of a new class of latent models in INLA that can be used to fit spatial econometric models directly to INLA. Gómez-Rubio and Rue (2018) [26] gave a new approach combining INLA and MCMC, namely MCMCINLA, making it possible to fit spatial econometric models. Gómez-Rubio et al. (2020) [27] further explored BMA by using INLABMA for multivariate posterior inference of spatial econometric models with good results.

Due to the limitation of random effects of spatial lag models, INLA currently only supports estimating spatial lag models in parametric form, and no scholars have yet used the method to estimate nonparametric spatial lag models. In this paper, we combine MCMC with INLA and propose MCMCINLA estimation of nonparametric spatial lag models with the help of penalized spline technique to explore the nonparametric relationship between covariates and response variables in varying coefficient spatial lag models. This estimation method can overcome the problem of long computation time of single MCMC and fill the gap that single INLA cannot estimate nonparametric spatial lag models, which can provide new ideas for statistical inference of nonparametric spatial econometric models.

According to statistics, the proportion of China’s population aged 65 and above has reached 13.5% by 2021, indicating that China has entered a deeply aging society [28]. In the context of an increasingly aging population, how to adjust the industrial structure to continue to effectively promote sustainable and healthy economic development has become a key issue for current research. In the past two years, a large number of scholars have explored the relationship between population aging, technological innovation, industrial structure and other factors and economic development using different methods, and some useful conclusions have been obtained. Song and Gao (2022) [29] explored the effects of population aging and private sector market entry on economic growth by constructing the benchmark regression econometric model and the synergistic effects econometric model. Li and Gao (2022) [30] studied the correlation between technological innovation and economic development in the perspective of population aging using the mediating effect and panel threshold effect model. Su (2021) [31] conducted an in-depth analysis of how population aging affects economic growth from the perspective of aggregate supply and aggregate demand based on the fixed effects econometric model. Li and Qin (2020) [32] explored the relationship between population aging, social security expenditures and economic development with the help of the dynamic panel data model.

However, in practical problems, the linear assumptions between economic growth and factors such as technological innovation, income expenditure, and industrial structure are often not satisfied, and simply constructing a spatial econometric model may not be sufficient to fully characterize the regression relationship among the variables and may easily ignore the key information. Based on the above considerations, this paper explores the economic development of 31 regions in mainland China from 2015 to 2020 using MCMCINLA, and constructs spatial lag models and varying coefficient spatial lag models successively for comparative analysis of influencing factors and spatial and temporal characteristic distribution analysis with population aging as a special variable, in order to further explore the hidden information and come up with economic development strategies and suggestions that are more in line with the realistic context of China.

## 2. Materials and methods

### 2.1 Data source and preprocessing

Through the China Statistical Yearbook (http://www.stats.gov.cn), we collect data on 15 socioeconomic indicators that can be used to reflect economic development in 31 regions of mainland China from 2015 to 2020 as the object of our study, and the specific indicators and corresponding variables for each indicator are shown in Table 1.

**Table 1:** Selected indicators and corresponding variables of China’s economic development research.

Variables	Indicators	Description
Economic development	Gross regional product (billion yuan)	Refers to the final results of production activities of all resident units in a region within a certain period of time
Urbanization rate	Share of year-end urban population by region (%)	Refers to the proportion of urban population to total population
Resident income	Per capita disposable income of residents by region (yuan)	Refers to the sum of household expenditure and savings available for final consumption
Resident consumption	Per capita consumption expenditure of residents by region (yuan)	Refers to the total expenditure of residents to meet the daily consumption needs of the family
Real estate development	Real estate development enterprises complete sets of residential sales (sets)	Refers to the total contract price of the house sold by the real estate development enterprise this year
	Real estate development enterprise main business income (billion yuan)	Refers to the total revenue from the main business of the real estate development enterprise this year
	Real estate development enterprises completed investment this year (billion yuan)	Refers to the total investment in housing construction projects and land development projects completed by real estate development enterprises this year
Foreign economy	Total import and export of goods by region (billion yuan)	Refers to the total amount of goods actually imported and exported to China’s customs territory
	Total import and export of goods of foreign-invested enterprises by region (million yuan)	Refers to the total amount of goods imported and exported in mainland China by foreign and Hong Kong, Macao and Taiwan legal persons
	Registration of foreign-invested enterprises by region at the end of the year (households)	Refers to the total number of enterprises registered in mainland China by foreign and Hong Kong, Macao and Taiwan legal persons
High-tech development	Industrial enterprises above the scale of invention patents by region (pieces)	Refers to the total number of invention patents of industrial enterprises of a certain scale
	Number of new product development projects in industrial enterprises above the scale by region (items)	Refers to the total number of new projects developed by industrial enterprises of a certain scale
	Technology market turnover by region (million yuan)	Refers to the total market turnover of high-tech products in various regions
	Number of major enterprises in national-level high-tech zones (pcs)	Refers to the total number of enterprises in national high-tech development zones nationwide
Population aging	Population age composition by region (persons)	Refers to the number of people over the age of 65 in each region

In view of the fact that the three variables of real estate development, foreign economy and high-tech development contain a large number of indicators, and there are certain correlations among the indicators with large differences in magnitudes, therefore, before the formal analysis, the data of the indicators involved in these three variables are firstly de-quantified and standardized, and multicollinearity diagnostics.

Abbreviate "real estate development" as A, the corresponding three indicators "sales of complete sets of residential units by real estate development enterprises", "main business income of real estate development enterprises" and "investment completed by real estate development enterprises in the current year" are *a*_1_, *a*_2_ and *a*_3_, respectively. Abbreviation "foreign economy" as B, the corresponding three indicators "total import and export of goods by region", "total import and export of goods by foreign-invested enterprises by region" and "registration of foreign-invested enterprises by region at the end of the year" are *b*_1_, *b*_2_ and *b*_3_, respectively. Abbreviation "high-tech development" as C, the corresponding four indicators "invention patents of industrial enterprises above the scale by region", "number of new product development projects of industrial enterprises above the scale by region", "technology market turnover by region" and "number of major enterprises in national high-tech zones" are *c*_1_, *c*_2_, *c*_3_ and *c*_4_, respectively. The results of the multicollinearity diagnostics of real estate development, foreign economy and high-tech development variables for 2015–2020 are shown in Tables 2–4.

**Table 2:** The results of the multicollinearity diagnostics of real estate development for 2015–2020.

Year	Indicator	Tol	VIF	Year	Indicator	Tol	VIF
2015	*a*_1_	0.234	4.281	2016	*a*_1_	0.163	6.121
	*a*_2_	0.105	9.533		*a*_2_	0.116	8.635
	*a*_*3*_	0.069	14.452		*a*_*3*_	0.055	18.234
2017	*a*_1_	0.171	5.849	2018	*a*_1_	0.295	3.389
	*a*_2_	0.096	10.386		*a*_2_	0.101	9.917
	*a*_*3*_	0.048	20.843		*a*_*3*_	0.077	12.995
2019	*a*_1_	0.470	2.127	2020	*a*_1_	0.206	4.861
	*a*_2_	0.056	17.979		*a*_2_	0.062	16.247
	*a*_*3*_	0.061	16.375		*a*_*3*_	0.041	24.574

**Table 3:** The results of the multicollinearity diagnostics of foreign economy for 2015–2020.

Year	Indicator	Tol	VIF	Year	Indicator	Tol	VIF
2015	*b*_1_	0.042	23.545	2016	*b*_1_	0040	24.740
	*b*_2_	0.050	19.872		*b*_2_	0.060	16.687
	*b*_3_	0.038	26.444		*b*_3_	0.035	28.269
2017	*b*_1_	0.044	22.616	2018	*b*_1_	0.062	16.069
	*b*_2_	0.085	11.826		*b*_2_	0.106	9.412
	*b*_3_	0.044	22.700		*b*_3_	0.076	13.093
2019	*b*_1_	0.074	13.542	2020	*b*_1_	0.072	13.976
	*b*_2_	0.126	7.935		*b*_2_	0.139	7.191
	*b*_3_	0.082	12.179		*b*_3_	0.081	12.318

**Table 4:** The results of the multicollinearity diagnostics of high-tech development for 2015–2020.

Year	Indicator	Tol	VIF	Year	Indicator	Tol	VIF
2015	*c*_1_	0.224	4.474	2016	*c*_1_	0.167	5.983
	*c*_2_	0.150	6.681		*c*_2_	0.129	7.742
	*c*_3_	0.032	31.078		*c*_3_	0.023	43.399
	*c*_4_	0.026	38.560		*c*_4_	0.019	52.907
2017	*c*_1_	0.108	9.231	2018	*c*_1_	0.095	10.539
	*c*_2_	0.106	9.447		*c*_2_	0.120	8.356
	*c*_3_	0.022	45.896		*c*_3_	0.067	14.871
	*c*_4_	0.017	57.574		*c*_4_	0.045	22.188
2019	*c*_1_	0.117	8.543	2020	*c*_1_	0.138	7.243
	*c*_2_	0.149	6.704		*c*_2_	0.165	6.055
	*c*_3_	0.065	15.324		*c*_3_	0.155	6.437
	*c*_4_	0.044	22.867		*c*_4_	0.123	8.135

Tolerance (TOL) or Variance Inflation Factor (VIF) are generally used as the goodness index to test the existence of collinearity. If Tol<0.1 or VIF>10, there is collinearity. It can be found from the results in Tables 2–4 that there are different degrees of Tol<0.1 or VIF>10 among the indicators in each year, indicating that the indicators of real estate development, foreign economy and high-tech development have collinearity, and further processing is required to make the research results more reliable.

In order to eliminate the multicollinearity among the indicators, we use principal component analysis to deal with it. The results of the principal component analysis of real estate development, foreign economy and high-tech development variables for 2015–2020 are shown in Tables 5–7.

**Table 5:** Principal component extraction of real estate development for 2015–2020.

Year	Indicator	Comp.1 loading	Comp.2 loading	Comp.3 loading	Comp.1 cumulative proportion	Comp.2 cumulative proportion	Comp.3 cumulative proportion
2015	*a*_1_	0.561	0.797	0.226	91.4%	98.5%	100%
	*a*_2_	0.578	-0.572	0.583			
	*a*_3_	0.593	-0.196	-0.781			
2016	*a*_1_	0.563	0.745	0.358	91.0%	98.8%	100%
	*a*_2_	0.570	-0.664	0.484			
	*a*_3_	0.598	0	-0.799			
2017	*a*_1_	0.559	0.762	0.327	90.2%	98.9%	100%
	*a*_2_	0.572	-0.639	0.514			
	*a*_3_	0.601	-0.101	-0.793			
2018	*a*_1_	0.556	0.818	0.150	90.6%	98.4%	100%
	*a*_2_	0.582	-0.512	0.632			
	*a*_3_	0.594	-0.264	-0.760			
2019	*a*_1_	0.532	0.845	0	86.6%	99.0%	100%
	*a*_2_	0.602	-0.335	-0.725			
	*a*_3_	0.595	-0.416	0.687			
2020	*a*_1_	0.559	0.805	0.198	92.3%	99.1%	100%
	*a*_2_	0.579	-0.550	0.602			
	*a*_3_	0.593	-0.222	-0.774			

**Table 6:** Principal component extraction of foreign economy for 2015–2020.

Year	Indicator	Comp.1 loading	Comp.2 loading	Comp.3 loading	Comp.1 cumulative proportion	Comp.2 cumulative proportion	Comp.3 cumulative proportion
2015	*b*_1_	0.577	0.577	0.597	98.0%	99.1%	100%
	*b*_2_	0.576	-0.796	0.185			
	*b*_3_	0.578	0.237	-0.781			
2016	*b*_1_	0.578	0.510	0.638	97.8%	99.2%	100%
	*b*_2_	0.576	-0.808	0.124			
	*b*_3_	0.579	0.295	-0.760			
2017	*b*_1_	0.579	0.408	0.706	97.2%	99.1%	100%
	*b*_2_	0.574	-0.819	0			
	*b*_3_	0.579	0.404	-0.708			
2018	*b*_1_	0.580	0.231	0.781	96.1%	98.6%	100%
	*b*_2_	0.574	-0.796	-0.191			
	*b*_3_	0.578	0.559	-0.595			
2019	*b*_1_	0.580	0.318	0.750	95.5%	98.4%	100%
	*b*_2_	0.573	-0.813	0			
	*b*_3_	0.579	0.487	-0.654			
2020	*b*_1_	0.581	0.316	0.750	95.3%	98.5%	100%
	*b*_2_	0.572	-0.814	-0.100			
	*b*_3_	0.579	0.487	-0.654			

**Table 7:** Principal component extraction of high-tech development for 2015–2020.

Year	Indicator	Comp.1 loading	Comp.2 loading	Comp.3 loading	Comp.4 loading	Comp.1 cumulative proportion	Comp.2 cumulative proportion	Comp.3 cumulative proportion	Comp.4 cumulative proportion
2015	*c*_1_	0.503	0.467	0.727	0	65.1%	96.4%	99.6%	100%
	*c*_2_	0.479	0.523	-0.677	0.197				
	*c*_3_	0.454	-0.603	0	0.654				
	*c*_4_	0.558	-0.379	-0.111	-0.730				
2016	*c*_1_	0.504	0.474	0.720	0	64.4%	97.3%	99.7%	100%
	*c*_2_	0.487	0.511	-0.690	0.159				
	*c*_3_	0.450	-0.600	0	0.661				
	*c*_4_	0.554	-0.393	0	-0.731				
2017	*c*_1_	0.511	0.465	0.716	0.103	65.7%	98.1%	99.7%	100%
	*c*_2_	0.493	0.504	-0.698	0.127				
	*c*_3_	0.440	-0.612	0	0.656				
	*c*_4_	0.550	-0.393	0	-0.736				
2018	*c*_1_	0.518	0.443	0.675	0.282	69.6%	97.7%	99.3%	100%
	*c*_2_	0.502	0.487	-0.715	0				
	*c*_3_	0.420	-0.665	-0.138	0.602				
	*c*_4_	0.551	-0.353	0.121	-0.747				
2019	*c*_1_	0.513	0.445	0.697	0.229	72.8%	97.3%	99.3%	100%
	*c*_2_	0.491	0.513	-0.703	0				
	*c*_3_	0.447	-0.643	-0.119	0.610				
	*c*_4_	0.544	-0.354	0	-0.757				
2020	*c*_1_	0.513	0.448	0.548	0.486	74.6%	96.0%	98.3%	100%
	*c*_2_	0.487	0.541	-0.594	-0.342				
	*c*_3_	0.478	-0.577	-0.426	0.508				
	*c*_4_	0.521	-0.418	0.406	-0.624				

According to the analysis results, the cumulative contribution rate of the first principal component of real estate development and foreign economy reached more than 85%, and the cumulative contribution rate of the first two principal components of high-tech development reached more than 95% in each year, so the first principal component of real estate development and foreign economy and the first two principal components of high-tech development are selected as the final variable data respectively. And then, the remaining five variables are also subjected to the de-quantile standardization operation to be used in the formal study.

### 2.2 Model building

Using the level of economic development as the response variable *y* and the seven variables of urbanization rate, resident income, resident consumption, real estate development, foreign economy, high-tech development and population aging as covariates *x*_*j*_, *j* = 1,2,…,7, constructing spatial lag model, namely, unvarying coefficient spatial lag model (denoted as "Model I"):

$$y={\left(I_{n}-\rho W\right)}^{-1}(x_{1}\beta_{1}+x_{2}\beta_{2}+x_{3}\beta_{3}+x_{4}\beta_{4}+x_{5}\beta_{5}+x_{6}\beta_{6}+x_{7}\beta_{7}+\varepsilon ),$$ (1)

where *n* = 186; *ρ* is the spatial autocorrelation parameter related to the spatial lag term, which can reflect the existence of spatial correlation between regions; *W* is the 186 × 186-dimensional spatial weight matrix between regions at different times; *β*_*j*_ is the regression coefficient of the covariate *x*_*j*_; the error term *ε* obeys a Gaussian distribution with mean 0 and diagonal variance-covariance matrix *σ*^2^*In*.

The assumption condition of linear relationship between covariates and response variables is relaxed on the basis of Model I. The variable coefficient term *β*_*j*_ (*U*) is introduced to consider spatial heterogeneity. In the context of China’s increasingly aging population, we pay more attention to the impact of population aging on economic development, and focus our research on the development and changes of the national economy with the deepening of aging. Taking population aging as a covariate *U* in the varying coefficient function, still using the level of economic development as the response variable *y*, and the remaining six variables as covariates *x*_*j*_, *j* = 1,2,…,6 to construct a varying coefficient spatial lag model to further explore the relationship between each influencing factor and economic development in the case of population aging. The varying coefficient spatial lag model (denoted as "Model II") is established as follows:

$$y={\left(I_{n}-\rho W\right)}^{-1}(x_{1}\beta_{1}(U)+x_{2}\beta_{2}(U)+x_{3}\beta_{3}(U)+x_{4}\beta_{4}(U)+x_{5}\beta_{5}(U)+x_{6}\beta_{6}(U)+\varepsilon ),$$ (2)

where *β*_*j*_ (*U*) = (*β*_*j*_ (*U*_1_), *β*_*j*_ (*U*_2_),…,*β*_*j*_ (*U*_*n*_)) ′ denotes the unknown nonparametric function on the covariate *U*. The remaining variables are defined in the same way as Model I.

### 2.3 MCMCINLA algorithm

The MCMCINLA algorithm was proposed by Gómez-Rubio and Rue in 2018 and can be used to fit spatial econometric models, linear regression models with covariates having missing data, Bayesian Lasso models and mixed models. The core idea of the algorithm is to divide the parameters to be estimated into two groups, one using the Metropolis-Hastings (MH) algorithm in MCMC and the other using the Bayesian Model Averaging (BMA) algorithm [33] in INLA, from which the estimates of all parameters in the model are obtained. Here, we use MH for the parameter *ρ* for estimation and BMA for the rest of the parameters.

Since the INLA algorithm mainly targets the model with a structured additive regression model with a latent random field of Gaussian Markov Random Fields (GMRF), it is first necessary to prove that the model conforms to the INLA framework before using MCMCINLA for estimating the varying coefficient spatial lag model. Using a Penalized spline method based on a cubic B spline basis with equidistant nodes to fit the varying coefficient term *β*_*j*_ (*U*) [34],

$$\beta_{j}(U)=\sum_{l=1}^{h}B_{l}^{\left(j\right)}\alpha_{l}^{\left(j\right)}=B^{\left(j\right)}\alpha^{\left(j\right)},$$

letting *z*_*j*_ = *x*_*j*_ *B*^(*j*)^, so that the varying coefficient spatial lag model can be rewritten as

$$\begin{array}{cc} y & =\rho Wy+\sum_{j=1}^{p}x_{j}\beta_{j}(U)+\varepsilon \\ & =\rho Wy\text{+}\sum_{j=1}^{p}z_{j}\alpha^{\left(j\right)}+\varepsilon , \end{array}$$ (3)

Achieving punishment by setting a priori, the prior for *α*^(*j*)^ is defined by the difference penalty, with first-order differences corresponding to first-order Random Walk and second-order differences corresponding to second-order Random Walk [35]. Here we use a second-order Random Walk with backward differences, so that *α*^(*j*)^ has a Gaussian prior with mean 0 and accuracy matrix Q, and its accuracy matrix Q is semipositive definite, such that *α*^(*j*)^ is an IGMRF [36]. Letting

$$\left\{\begin{array}{l} y=x+\xi \\ x={\left(I_{n}-\rho W\right)}^{-1}\left(Z\alpha +\varepsilon \right) \end{array}\right.,$$ (4)

where *ξ* is the perturbation term added to the model. According to the previous analysis, it is known that *α* has a Gaussian prior with mean 0 and accuracy matrix Q, and *ε* obeys a Gaussian distribution with mean 0 and accuracy matrix *τI*_*n*_. Therefore, (*x*, *α*) is a GMRF with mean 0 and precision matrix *P* (structure as follows), so the model conforms to the INLA framework and can be solved using MCMCINLA. Only the main results are shown here, and the detailed proof procedure is shown in S1 Appendix.


$$\begin{array}{cc} P & =\left(\begin{array}{cc} T & -T{\left(I_{n}-\rho W\right)}^{-1}Z\\ -Z\prime {\left(I_{n}-\rho W\prime \right)}^{-1}T & Q+\tau Z\prime Z \end{array}\right)\\ & =\left(\begin{array}{cc} \tau (I_{n}-\rho W\prime )(I_{n}-\rho W) & -\tau (I_{n}-\rho W\prime )Z\\ -\tau Z\prime (I_{n}-\rho W) & Q+\tau Z\prime Z \end{array}\right). \end{array}$$

It is worth noting that in the expansion of the varying coefficient term *β_j_* (*U*) using the B spline basis function, its choice of both the number and location of nodes is very sensitive [37]. To solve this selection problem, Eilers and Marx (1996) [38] suggested using a moderate number of nodes and defining a roughness penalty based on the difference of adjacent B spline coefficients to ensure that the fitted curve is sufficiently smooth, which here corresponds to using a second-order Random Walk (RW2) prior for the penalty. The number of nodes is generally selected using goodness-of-fit metrics such as AIC, BIC, and DIC, and here we use the DIC to select the number of nodes. Another noteworthy issue is that the mean of the varying coefficient term *β_j_* (*U*) is generally unidentifiable [39]. To ensure identifiability, it is required that *β_j_* (*U*) is constrained to have zero mean, i.e., it satisfies $\frac{1}{range(U)}\int \beta_{j}\left(U\right)dU=0$, and thus *β_j_* (*U*) can be included in the estimation at each iteration by MCMC centered on its mean.

The estimation process of fitting the varying coefficient spatial lag model using the MCMCINLA algorithm is performed in four main steps as follows. All calculations are done in the R-INLA (http://www.r-inla.org) environment in R.

1. P spline transformation. The curve is fitted using a cubic B spline basis function and the RW2 prior *f(U*, *model = "rw2")* is set as a penalty for the model, at which time the parameters to be estimated in the model are the parameter *ρ*, the hyperparameter *τ* and the parameter *α*.
2. Estimation of *ρ* using the MH algorithm. The process is carried out in three main steps as follows.

Step1: Assume that starting from the initial point *ρ*^(1)^ = 0, the model is fitted conditionally with *ρ*^(1)^ to obtain *π*(*y*|*ρ*^(1)^), *π*(*τ*|*y*,*ρ*^(1)^) and *π*(*α*|*y*, *ρ*^(1)^);

Step2: Use the MH algorithm to sample from the posterior of *ρ*, propose a new point ρ* for *ρ* by proposing the distribution *q*(∙│*ρ*^(*j*−1)^), fit the model conditionally on ρ* to obtain *π*(*y*|ρ*), *π*(*τ*|*y*,ρ*) and *π*(*α*│*y*,ρ*), and calculate *π*(ρ*), *q*(ρ*|*ρ*^(j)^) and *q*(*ρ*^(*j*)^)|ρ*);

Step3: Calculate the acceptance probability *P* of ρ* and determine whether the proposal is acceptable or not, where

$$P=\text{min}\{1,\frac{\pi (\tau |y,\rho^{*})\pi (\alpha |y,\rho^{*})\pi (y|\rho^{*})\pi (\rho^{*})q(\rho^{\left(j\right)}|\rho^{*})}{\pi (\tau |y,\rho^{\left(j\right)})\pi (\alpha |y,\rho^{\left(j\right)})\pi (y|\rho^{\left(j\right)})\pi (\rho^{\left(j\right)})q(\rho^{*}|\rho^{\left(j\right)})}\},$$ (5)

if the proposal is accepted, then *ρ*^(*j*+1)^ = ρ* with *π*(*τ*│*y*,*ρ*^(*j*+1)^) = *π*(*τ*│*y*,ρ*), *π*(*τ*│*y*,*ρ*^(*j*+1)^) = *π*(*τ*│*y*,ρ*), *π*(*α*│*y*,*ρ*^(*j*+1)^) = *π*(*α*│*y*,ρ*); otherwise, *ρ*^(*j*+1)^ = *ρ*^(*j*)^, and *π*(*τ*│*y*,*ρ*^(*j*+1)^) = *π*(*τ*│*y*,*ρ*^(*j*)^), *π*(*α*│*y*,*ρ*^(*j*+1)^) = *π*(*α*│*y*,*ρ*^(*j*)^). This iterative process is executed until the end of the estimation.

3. Estimation of *τ* and *α* using the BMA algorithm. For the hyperparameter *τ* and the parameter *α*, the conditional margins *π*(*τ*│*y*,*ρ*^(*j*)^) and *π*(*α*│*y*,*ρ*^(*j*)^) generated by the MH algorithm in each iteration can be obtained using BMA, and further the posterior margins of0 *τ* and *α* are derived by integrating over *ρ*, namely

$$\pi (\tau |y)=\int \pi (\tau |\rho ,y)\pi (\rho |y)d\rho =\frac{1}{N}\sum_{j=1}^{N}\pi (\tau |y,\rho^{\left(j\right)}).$$ (6)

$$\pi (\alpha |y)=\int \pi (\alpha |\rho ,y)\pi (\rho |y)d\rho =\frac{1}{N}\sum_{j=1}^{N}\pi (\alpha |y,\rho^{\left(j\right)}).$$ (7)

4. Estimation of *β*_*j*_ (*U*). The model fitted using MCMCINLA is the model fitted by P spline, which is directly obtained as an estimate about *α*^(*j*)^. Therefore, it is finally necessary to multiply it with the design matrix *B*^(*j*)^ to obtain an estimate of the varying coefficient term *β*_*j*_ (*U*) using *β*_*j*_ (*U*) = B^(*j*)^α^(*j*)^.

## 3. Results analysis

### 3.1 Model selection

Deviance information criterion (DIC) and Widely applicable information criterion (WAIC) are two commonly used metrics to evaluate the fitting effectiveness of Bayesian models, and in general, the smaller the value of DIC and WAIC, the better the fit of the model. From the fitting results in Table 8, it can be found that the DIC and WAIC value of the two models are very small, and the value of Model II is smaller than that of Model I, indicating that the fitting effect of Model II is better, which is more suitable for studying the dynamic changes of economic development.

**Table 8:** Fitting evaluation results of Model I and Model II.

Model	DIC	WAIC
Model I	-2076.16	-2133.23
Model II	-2144.19	-2283.90

### 3.2 Analysis of factors influencing with spatial lag model

**Fig 1:**
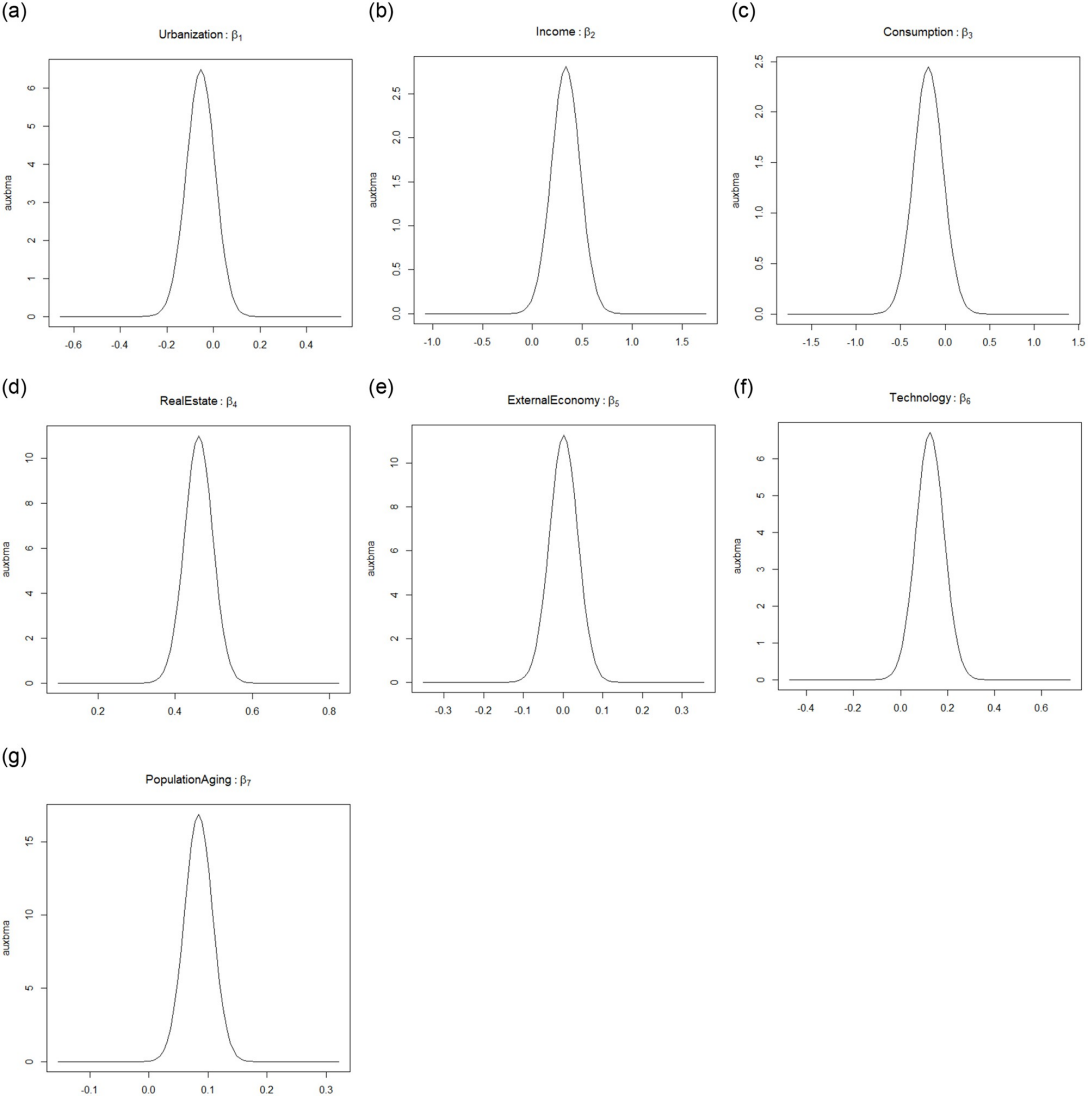
Regression coefficients of each influence factor of the spatial lag model.

The Model I is fitted using MCMCINLA, and the estimated values of the spatial autocorrelation parameters and the posterior estimates of the regression coefficients of each influencing factor are obtained as shown in Table 9, and the regression coefficients of each influencing factor are plotted as shown in Fig 1. As estimated by MCMCINLA, the spatial autocorrelation parameter is *ρ* = 0.5946 (95%CI:0.3522~0.8450), indicating that there is a significant spatial correlation between the economic development of provinces. To a certain extent, the economic development of a region also stimulates the economic growth of the surrounding regions. From the results in Table 9, it can be found that the 95% credible intervals for urbanization rate, resident consumption and foreign economy contain 0, indicating that these three factors have no effect on the economic development of the region. The posterior mean of resident income, real estate development, high-tech development and population aging are 0.334, 0.460, 0.124 and 0.084, respectively, indicating that these four factors have significant positive effects on the economic development of the region, and real estate development has the strongest contribution to the economic development of the whole region.

**Table 9:** Posterior estimates of regression coefficients of each influence factor in spatial lag model.

Variables	Mean	Variance	95% Credible interval
Urbanization rate	-0.055	0.061	(-0.177,0.064)
Resident income	0.334	0.142	(0.052,0.612)
Resident consumption	-0.188	0.163	(-0.509,0.131)
Real estate development	0.460	0.036	(0.388,0.531)
Foreign economy	0.001	0.035	(-0.068,0.070)
High-tech development	0.124	0.059	(0.007,0.241)
Population aging	0.084	0.023	(0.037,0.130)

### 3.3 Analysis of factors influencing with varying coefficient spatial lag model

**Fig 2:**
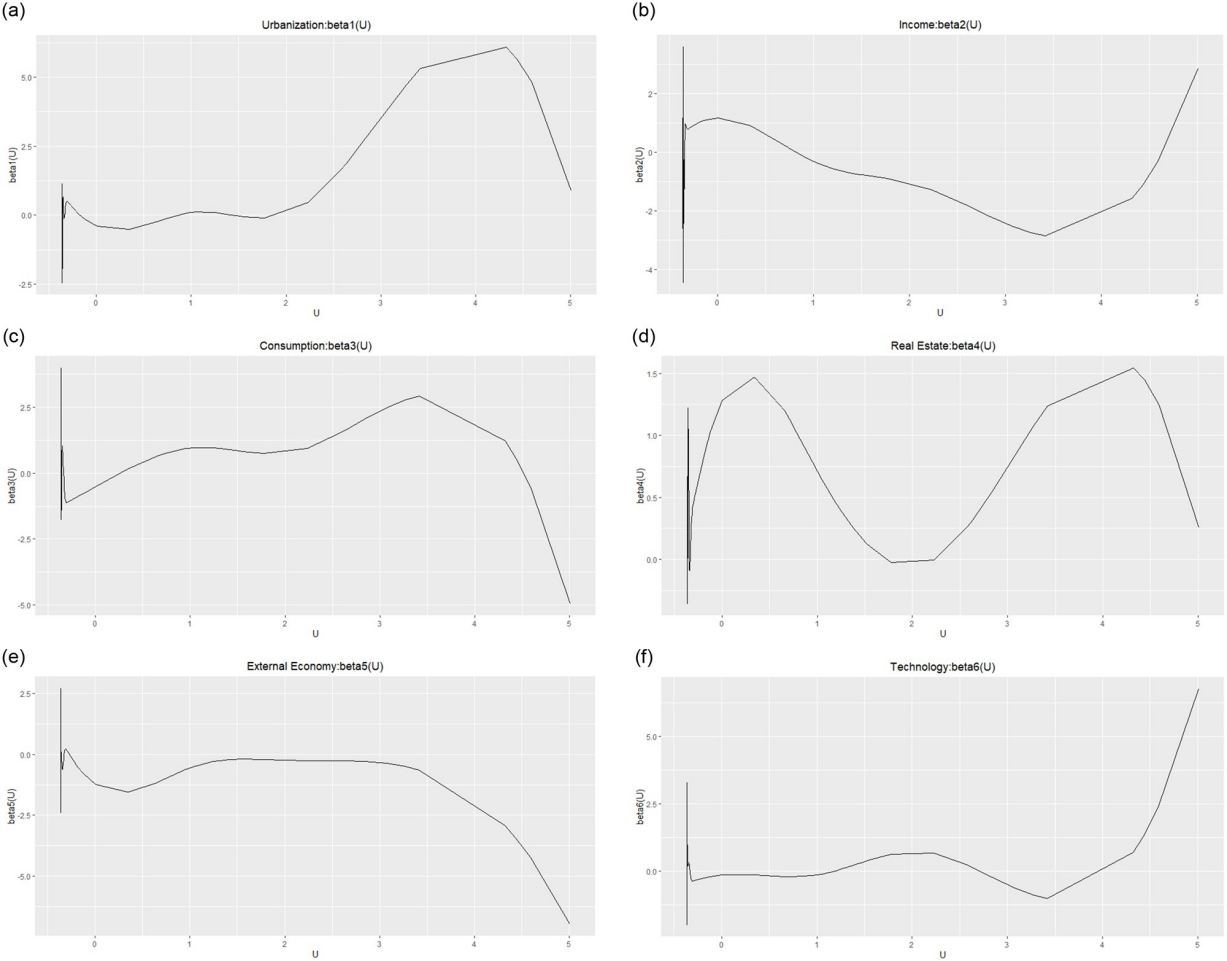
Regression coefficients of each influence factor of the varying coefficient spatial lag model.

The Model II is fitted using MCMCINLA, and the estimated values of the spatial autocorrelation parameters and the posterior estimates of the regression coefficients of each influencing factor are obtained as shown in Table 10, and the regression coefficients of each influencing factor are plotted as shown in Fig 2. At this point, the spatial autocorrelation parameter is *ρ* = 0.2745 (95%CI:0.0816~0.4312). The 95% credible interval for resident consumption contains 0, indicating that resident consumption has no impact on economic development. The factor that has a significant negative effect on economic development is the foreign economy, with a posteriori mean of -0.263. The factors that contribute significantly to economic development are urbanization rate, resident income, real estate development and high-tech development, with posteriori means of 0.143, 0.127, 0.256 and 0.357, respectively. At the same time, it can be found that in the context of aging, high-tech development has replaced real estate development as the most mobilizing factor for regional economic development.

**Table 10:** Posterior estimates of regression coefficients of each influence factor in the varying coefficient spatial lag model.

Variables	Mean	Variance	95% Credible interval
Urbanization rate	0.143	0.054	(0.035,0.252)
Resident income	0.127	0.056	(0.015,0.237)
Resident consumption	-0.153	0.128	(-0.409,0.103)
Real estate development	0.256	0.043	(0.169,0.342)
Foreign economy	-0.263	0.039	(-0.340,-0.185)
High-tech development	0.357	0.060	(0.237,0.477)

According to Fig 2, it is possible to further explore the changes of economic development and each factor in the scenario of increasing population aging in Chinese society. It is clear from the figure that there is not a direct linear relationship between the influencing factors and economic development. The urbanization rate curve is basically located in the positive half-axis of the y-axis, indicating that the urbanization process plays a certain role in promoting the development of regional economy, and with the gradual increase of aging, the promotion role begins to gradually weaken after reaching a certain peak. The main reason for this change is that the urbanization process can promote the influx of rural population to the cities to provide more labor for the industrial development of the cities [40], while the increasing aging problem makes the industrial development generate the problem of insufficient labor supply, which leads to some impact on the economic development rate of the region. The vast majority of the income curve lies on the positive half-axis of the y-axis, indicating that the income level of residents also plays a certain role in promoting the development of the regional economy, and with the gradual aggravation of aging, the role of income in promoting economic development is first decreasing and then increasing. The real estate development curves are basically located in the positive half-axis of the y-axis, indicating that the development of real estate has a certain role in promoting the development of the regional economy, and it is most fluctuated by the influence of aging. The main reason is that the construction of real estate requires a large amount of labor force, and the increasing proportion of the aging population directly reduces the main source of engineering construction, which has a certain impact on the development of the real estate industry and the economic development of the region. The foreign economy curve basically lies in the negative half-axis of the y-axis, indicating that the development of external economic trade does not have a catalytic effect on regional economic growth, and with the gradual increase of aging, the hindering effect of foreign economy on economic development becomes more and more obvious. The main reason for this phenomenon is the decline in the supply of young adult labor after reaching its peak in the context of an aging population, which has led some foreign-invested companies to relocate their factories to regions with a large pool of low-cost labor, such as India [41], thus hampering foreign trade and economic development. The curve of high-tech development basically lies in the positive half-axis of the y-axis, indicating that the development of high-tech plays a certain role in promoting the development of regional economy, and the promotion role gradually increases with the gradual aggravation of aging. The aging population has made people realize that it is difficult to continue to promote rapid economic growth by relying on labor-intensive industries, so they have started to adjust the industrial structure and use high-tech intelligent equipment to replace labor, thus alleviating the economic impact caused by the lack of labor supply [42] and enabling the sustainable and healthy development of the regional economy.

### 3.4 Analysis of spatial and temporal characteristics with varying coefficient spatial lag model

**Fig 3:**
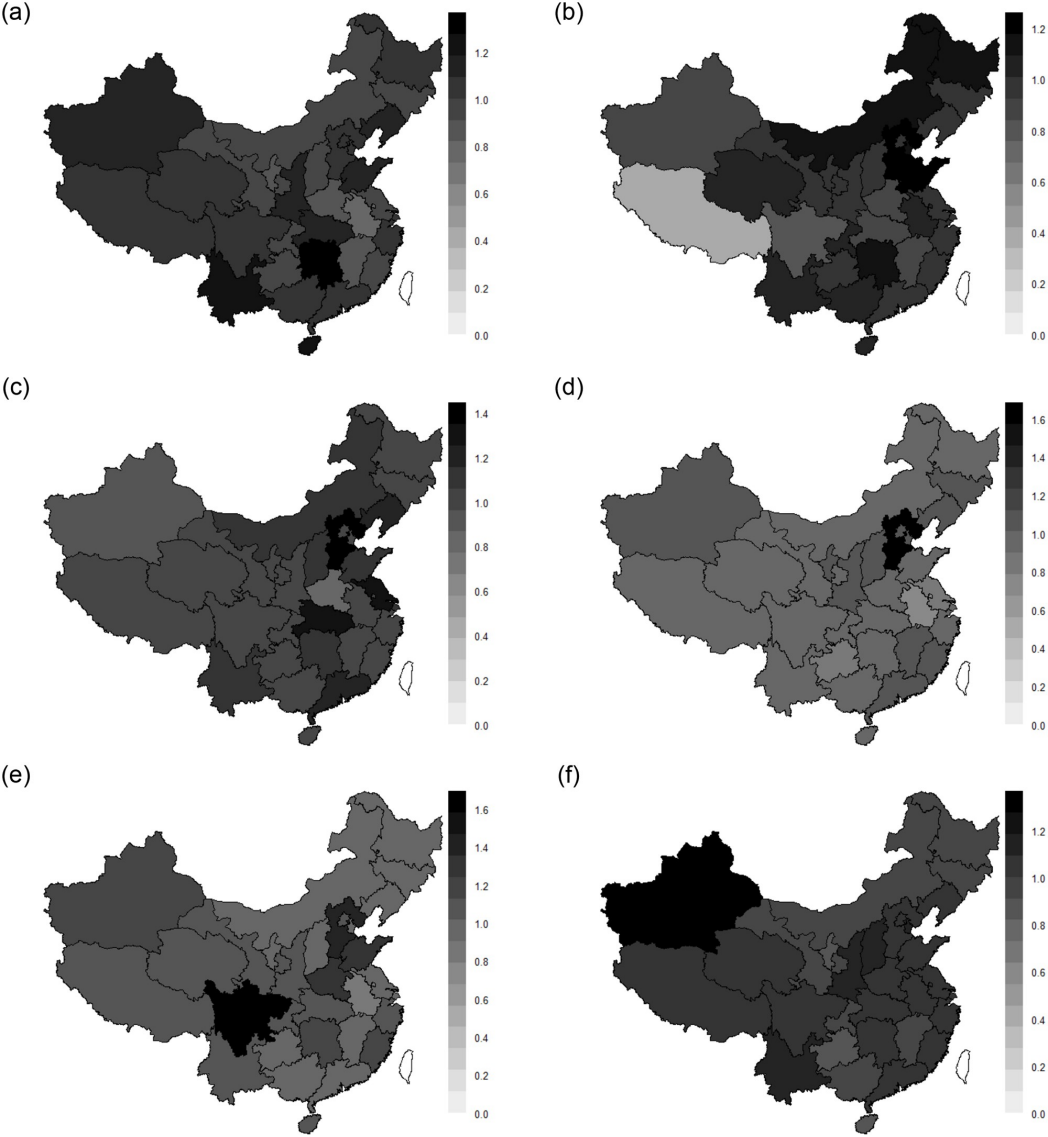
Spatial and temporal distribution of China’s economic development from 2015 to 2020.

The posterior mean of population aging variables are obtained using MCMCINLA and the spatial and temporal distribution of China’s economic development from 2015 to 2020 is plotted as shown in Fig 3. It can be found that from a temporal perspective, the economic development of each region during 2015–2019 has continued to improve, and by 2019 there have been a number of regions such as Hebei and Sichuan where the posterior mean of economic development has reached more than 1.4, while thereafter, the COVID-19 began to appear in Wuhan City, Hubei Province at the end of 2019, and under the impact of the epidemic, the economic level of each region in the country in 2020 has seen a significant decline, reaching a maximum of only 1.27. From a spatial perspective, the development of China’s economy has some spatial variability, with the economic development level of the eastern provinces being significantly higher than that of the western provinces. And with the deepening of aging in recent years, Hebei, Sichuan, Hubei and Jiangsu still lead the country in terms of economic level. The "Beijing-Tianjin-Hebei" city circle with Beijing and Tianjin as the center and Hebei as the joint venture, the "Sichuan-Chongqing" city cluster with Sichuan and Chongqing as the center, and the "Yangtze River Delta" economic zone built around the Yangtze River basin have attracted many young people from other provinces to move to them, enabling the regional economy to continue to grow despite the increasing aging population. In addition, according to the spatial distribution map of China’s economic development in 2020, it can be found that Xinjiang may become a potential region for future economic growth. It can draw on the development experience of urban agglomerations to build a network of cities in the northwest, shrink the economic differences between the east and west, and promote further economic development in the northwest. From the perspective of space-time, the gap between the rich and the poor has gradually narrowed, the level of economic development has become more balanced, and the national economic status has tended to be stable. The deepening of population aging has prompted many enterprises to start the transformation from traditional industries to high-tech industries, and through the construction of urban agglomerations, "from point to area", drive the economic development of many regions, and effectively achieve common prosperity.

## 4. Discussion

By using MCMCINLA to fit the spatial lag model with unvarying coefficients and the spatial lag model with varying coefficients respectively, it can be found that both models conclude that resident income, real estate development and high-tech development will positively contribute to regional economic growth.

However, the comparison also reveals the differences between the two models. After considering the study object in a nonparametric framework, the spatial autocorrelation parameter of the model is significantly lower. The assumption of linear relationship between variables in the classical spatial lag model affects the spatial relationship to a certain extent, making the estimation of spatial correlation high; however, in the context of varying coefficients, this problem can be corrected, making the estimation of spatial autocorrelation parameters in the model more accurate.

After considering the study object in a nonparametric framework, the effect of some of the influencing factors on the response variable has changed, making the results more relevant to reality. For example, the effects of urbanization rate and foreign economy on economic growth change from no effect to positive and negative effects, respectively. To a certain extent, promoting urbanization can promote the development of industry, which in turn promotes regional economic growth; the aging population makes foreign companies shift more to countries with more cheap labor, leading to some restrictions on the development of foreign economy, which is consistent with reality.

After considering the study object in a nonparametric framework, it is possible to dig deeper into the changing relationships and evolutionary trends among variables. Under the condition of constant coefficients, we can only derive the one-to-one correspondence between each influencing factor and the response variable; while in the context of varying coefficients, we can further obtain the trend of the remaining variables and the response variable as one variable changes, so as to make an accurate study of future development and realize one-to-many.

After considering the study object in a nonparametric framework, important variables can be more precisely located to provide strategic references for future development. For example, after setting the factor of population aging as a covariate of the varying coefficient function, the most influential factor on economic development changes from real estate development to high-tech development, which is more suitable for the current economic and social reality in China, so the adjustment of industrial structure can be focused on high-tech, automatic and intelligent system construction in the future.

## 5. Conclusion

Classical spatial lag models often require linear assumption modeling, while they can also explore the correlation characteristics of spatial data themselves, they are not sufficient to fully characterize the regression relationships between variables in a linear framework alone. This paper explores the nonparametric relationships between covariates and response variables in the varying coefficient spatial lag model based on the P spline, so as to make up for the shortcomings of the single linear regression of the classical spatial lag model and make it more practical. In terms of parameter estimation, we propose the MCMCINLA estimation method for nonparametric spatial lag models, which overcomes the problem of long computation time of single MCMC algorithm and fills the gap that single INLA algorithm cannot estimate nonparametric spatial lag models, and can provide new ideas for statistical inference. In addition, the economic development data of 31 regions in China over 6 years are used as examples to explore the issue of the influencing factors and spatial and temporal distribution of economic development under the classical spatial lag model and under the varying coefficient spatial lag model, respectively. In contrast, it is found that the varying coefficient spatial lag model can more fully explore the data information more in line with the actual background, which can provide judgment and reference for the development trend and strategy of the economic market.

In view of the high complexity of nonparametric spatial lag model itself, compared with MCMC algorithm, MCMCINLA has greatly reduced the operation time, but due to the existence of MH sampling in the algorithm, the algorithm still has the problem of long calculation time in the face of high-dimensional data, which is more suitable for small sample analysis. In addition, this paper mainly analyzes longitudinal data by setting the coefficient function as an unknown function of a covariate. In the future, it can be extended to spatial panel data, and the coefficient function can be set as a function form of spatial position coordinates to conduct more in-depth research on spatial distribution and spatiotemporal distribution.

## Supporting information

S1 Data Socioeconomic data files of various regions in mainland China from 2015 to 2020. (ZIP)

S1 Appendix Proof of GMRF structure for the varying coefficient spatial lag model. (PDF)

S2 Appendix R code for analysis of China’s economic development under the classical spatial lag model and varying coefficient spatial lag model. (PDF)
