# Supplementary material for: MCMCINLA estimation of varying coefficient spatial lag model—A study of China’s economic development in the context of population aging
Source: PLoS One. 2023 May 15;18(5):e0279504. doi: 10.1371/journal.pone.0279504 (PMC10184945; doi:10.1371/journal.pone.0279504)
Supplement: S1 Appendix — (PDF) [file pone.0279504.s002.pdf]

S1 Appendix. Proof of GMRF Structure for the Varying Coefficient Spatial Lag Model.

For the varying coefficient spatial lag model constructed in this paper,

$$y = \rho Wy + \sum_{j=1}^{p} x_j \beta_j(U) + \varepsilon, \varepsilon \sim N(0, \sigma^2 I_n). \tag{1}$$

The varying coefficient term $\beta_j(U)$ is fitted using a Penalized spline method based on the B spline basis. Approximating $\beta_j(U)$ by a dth B spline with equidistant nodes $U_{min} < t_1 < \cdots < t_r < U_{max}$:

$$\beta_j(U) = \sum_{l=1}^{h} B_l^{(j)} \alpha_l^{(j)},$$

where $h = d + r + 1$ is the degree of freedom and $B_l^{(j)}$ is the dth B spline basis function. Writing the expansion in the form of matrix multiplication, we have:

$$\begin{aligned}\beta_j(U) &= \sum_{l=1}^{h} B_l^{(j)} \alpha_l^{(j)} \\ &= B_1^{(j)} \alpha_1^{(j)} + B_2^{(j)} \alpha_2^{(j)} + \cdots + B_h^{(j)} \alpha_h^{(j)} \\ &= \begin{pmatrix} B_1^{(j)} & & & \\ & B_2^{(j)} & & \\ & & \ddots & \\ & & & B_h^{(j)} \end{pmatrix} \begin{pmatrix} \alpha_1^{(j)} \\ \alpha_2^{(j)} \\ \vdots \\ \alpha_h^{(j)} \end{pmatrix} \\ &= B^{(j)} \alpha^{(j)},\end{aligned} \tag{2}$$

where $B^{(j)}$ is the $n \times h$ dimensional design matrix and $\alpha^{(j)}$ is the h dimensional column vector. Let $z_j = x_j B^{(j)}$, so that this varying coefficient spatial lag model can be rewritten as:

$$\begin{aligned} y &= \rho Wy + \sum_{j=1}^{p} x_j \beta_j(U) + \varepsilon \\ &= \rho Wy + \sum_{j=1}^{p} x_j B^{(j)} \alpha^{(j)} + \varepsilon \\ &= \rho Wy + \sum_{j=1}^{p} z_j \alpha^{(j)} + \varepsilon \\ &= \rho Wy + z_1 \alpha^{(1)} + z_2 \alpha^{(2)} + \cdots + z_p \alpha^{(p)} + \varepsilon. \end{aligned} \tag{3}$$

In the expansion of the varying coefficient term $\beta_j(U)$ using the B spline basis function, it is

very sensitive to the choice of both the number and location of nodes. To solve this selection problem, Eilers and Marx (1996) suggested using a moderate number of nodes and defining the roughness penalty based on the difference of adjacent B spline coefficients to ensure that the fitted curve is sufficiently smooth. Here, following the suggestion of Eilers et al. its penalty likelihood is obtained as

$$L = l(y, \alpha^{(1)}, \alpha^{(2)}, \cdots, \alpha^{(p)}) - \lambda_1 \sum_{l=k+1}^{h} (\Delta^k \alpha_l^{(1)})^2 - \cdots - \lambda_p \sum_{l=k+1}^{h} (\Delta^k \alpha_l^{(p)})^2,$$

where Δ^k denotes the kth order difference operator.

The prior of $\alpha^{(j)}$ is defined by the difference penalty, with first-order difference corresponding to first-order Random Walk and second-order difference corresponding to second-order Random Walk. In this paper, we choose to use the second-order Random Walk with backward differencing, then its corresponding second-order differencing is:

$$\Delta^2 \alpha_l^{(j)} = \alpha_l^{(j)} - 2\alpha_{l-1}^{(j)} + \alpha_{l-2}^{(j)} \sim N(0, \tau^{-1}). \quad (4)$$

Thereby, the joint density of $\alpha^{(j)}$ is:

$$\begin{aligned} \pi(\alpha^{(j)}) &\propto \tau^{(n-2)/2} \exp(-\frac{\tau}{2} \sum (\Delta^2 \alpha_l^{(j)})^2) \\ &\propto \tau^{(n-2)/2} \exp(-\frac{\tau}{2} \sum (\alpha_l^{(j)} - 2\alpha_{l-1}^{(j)} + \alpha_{l-2}^{(j)})^2) \quad (5) \\ &\propto \tau^{(n-2)/2} \exp(-\frac{1}{2} \alpha^{(j)T} Q \alpha^{(j)}), \end{aligned}$$

where $Q = \tau R$, $R = \begin{pmatrix} 1 & -2 & 1 & & & & & & \\ -2 & 5 & -4 & 1 & & & & & \\ 1 & -4 & 6 & -4 & 1 & & & & \\ & 1 & -4 & 6 & -4 & 1 & & & \\ & & \ddots & \ddots & \ddots & \ddots & \ddots & & \\ & & & 1 & -4 & 6 & -4 & 1 & \\ & & & & 1 & -4 & 6 & -4 & 1 \\ & & & & & 1 & -4 & 5 & -2 \\ & & & & & & 1 & -2 & 1 \end{pmatrix}$.

Thus, $\alpha^{(j)}$ has a Gaussian prior with mean 0 and accuracy matrix Q. And here the accuracy matrix Q is semipositive definite with rank $n-2$, such that $\alpha^{(j)}$ is an IGMRF. The penalty is achieved by setting the prior, which corresponds to using the RW2 prior here.

By shifting the terms of Eq (3), it is rewritten in the following form:

$$\begin{aligned} y &= (I_n - \rho W)^{-1}(z_1\alpha^{(1)} + z_2\alpha^{(2)} + \cdots + z_p\alpha^{(p)} + \varepsilon) \\ &= (I_n - \rho W)^{-1}(Z\alpha + \varepsilon). \end{aligned} \tag{6}$$

Let

$$\begin{cases} y = x + \xi \\ x = (I_n - \rho W)^{-1}(Z\alpha + \varepsilon) \end{cases}, \tag{7}$$

where ξ is the perturbation term added to the model, and it is known from the previous analysis that α has a Gaussian prior with mean 0 and accuracy matrix Q, and ε obeys a Gaussian distribution with mean 0 and accuracy matrix τI_n. R-INLA is mainly computed for the joint distribution of x and α, that is,(x, α). Therefore, to prove that the model can be computed using INLA, it is necessary to verify that the model obeys the GMRF structure, in other words, it is sufficient to verify that (x, α) is a GMRF with a sparse accuracy matrix.

According to Bayes' theorem, we have:

$$\pi(x, \alpha) = \pi(x \mid \alpha)\pi(\alpha).$$

Assuming that the joint distribution is Gaussian, then its conditional distribution $(x|\alpha)$ is

also Gaussian, then

$$M = E(x \mid \alpha) = (I_n - \rho W)^{-1} Z\alpha, \tag{8}$$

$$\begin{aligned}\operatorname{var}(x \mid \alpha) &= \operatorname{var}((I_n - \rho W)^{-1} Z\alpha + (I_n - \rho W)^{-1}\varepsilon \mid \alpha) \\ &= (I_n - \rho W)^{-1} \operatorname{var}(\varepsilon \mid \alpha)((I_n - \rho W)^{-1})' \\ &= (I_n - \rho W)^{-1} \frac{1}{\tau} I_n ((I_n - \rho W)^{-1})' \\ &= \frac{1}{\tau}(I_n - \rho W)^{-1}(I_n - \rho W')^{-1},\end{aligned}$$

$$T = \operatorname{Prec}(x \mid \alpha) = \frac{1}{\operatorname{var}(x \mid \alpha)} = \tau(I_n - \rho W')(I_n - \rho W), \tag{9}$$

the precision matrix T is symmetric and sparse. Thereby, the joint distribution of x and α is:

$$\begin{aligned}\pi(x,\alpha) &= \pi(x \mid \alpha)\pi(\alpha) \\ &\propto \exp\{-\frac{1}{2}(x-M)'T(x-M)\}\exp\{-\frac{1}{2}(\alpha-0)'Q(\alpha-0)\} \\ &\propto \exp\{-\frac{1}{2}(x-M)'T(x-M)\}\exp\{-\frac{1}{2}\alpha'Q\alpha\} \\ &\propto \exp\{-\frac{1}{2}(x'Tx - x'TM - M'Tx + M'TM + \alpha'Q\alpha)\} \\ &\propto \exp\{-\frac{1}{2}(x,\alpha)'P(x,\alpha)\},\end{aligned} \tag{10}$$

where P is the precision matrix of (x,α) with the structure of

$$\begin{aligned}P &= \begin{pmatrix} T & -T(I_n - \rho W)^{-1}Z \\ -Z'(I_n - \rho W')^{-1}T & Q + \tau Z'Z \end{pmatrix} \\ &= \begin{pmatrix} \tau(I_n - \rho W')(I_n - \rho W) & -\tau(I_n - \rho W')Z \\ -\tau Z'(I_n - \rho W) & Q + \tau Z'Z \end{pmatrix}.\end{aligned}$$

And,

$$\begin{aligned}x'TM &= x'\tau(I_n - \rho W')(I_n - \rho W)(I_n - \rho W)^{-1}Z\alpha \\ &= \tau x'(I_n - \rho W')Z\alpha,\end{aligned}$$

$$M'Tx = (x'TM)' = \tau\alpha'Z'(I_n - \rho W)x,$$

$$\begin{aligned}M'TM &= \tau\alpha'Z'(I_n - \rho W')^{-1}(I_n - \rho W')(I_n - \rho W)(I_n - \rho W)^{-1}Z\alpha \\ &= \tau\alpha'Z'Z\alpha.\end{aligned}$$

Thus,

$$E(x,\alpha)=0. \tag{11}$$

Consequently, (x,α) is a GMRF with mean 0 and accuracy matrix P. Therefore, the model conforms to the INLA framework.
