# Supplementary material for: MCMCINLA estimation of varying coefficient spatial lag model—A study of China’s economic development in the context of population aging
Source: PLoS One. 2023 May 15;18(5):e0279504. doi: 10.1371/journal.pone.0279504 (PMC10184945; doi:10.1371/journal.pone.0279504)
Supplement: S2 Appendix — (PDF) [file pone.0279504.s003.pdf]

S2 Appendix. R code for analysis of China's economic development under the Classical Spatial Lag Model and Varying Coefficient Spatial Lag Model.

1.R code for analysis of China's economic development under the Classical Spatial Lag Model (Section 3.2 including Section 3.1)

```
library(INLA)
library(spdep)
library(INLABMA)
library(rgdal)
install.packages("spatialreg")
library(spatialreg)
library(spatstat)
library(sp)
library(maptools)
library(parallel)

data_China.economy <- data.frame(read.csv("C:/Users/teng/Desktop/paper/finaldata2.csv", head = T, sep = ","))

library(ggplot2)
library(maptools)
#China.gen<-readShapePoly("Export_Output.shp")
China.gen<-st_read("C:/Users/teng/Desktop/paper /Export_Output.shp")
China.gen$NAME

China_nb <- spdep::poly2nb(China.gen,queen=T)
censored1 <- China.gen$NAME== "台湾"
Chinadata1 <- China.gen[!censored1,]
censored2 <- Chinadata1$NAME== "香港"
Chinadata2 <- Chinadata1[!censored2,]
censored3 <- Chinadata2$NAME== "澳门"
Chinadata3 <- Chinadata2[!censored3,]
total1<-rbind(Chinadata3,Chinadata3)
total2<-rbind(total1,total1)
total3<-rbind(Chinadata3,total2)
total4<-rbind(Chinadata3,total3)
China_nb <- spdep::poly2nb(total4,queen=T)
China_lw <- spdep::nb2listw(China_nb, style="W")
China_W <- as(as_dgRMatrix_listw(China_lw), "CsparseMatrix")

N=186
data=data_China.economy
x1<-scale(data$x1)
x2<-scale(data$x2)
```

```
x3<-scale(data$x3)
x4<-data$x4
x5<-data$x5
x6<-data$x6
x7<-scale(data$x7)
y<-scale(data$y)

data2=data.frame(x1=x1,x2=x2,x3=x3,x4=x4,x5=x5,x6=x6,x7=x7,y=y,idx=1:N)
form <- y~ -1+ x1 + x2 + x3 + x4 + x5+ x6 + x7

zero.variance = list(prec=list(initial = 15, fixed=TRUE))

fit.inla <- function(data, rho) {
  #, form = form, W = W,
  #    zero.variance = zero.variance) {
  res <- slm.inla(form, d = data2, W = China_W, rho = rho,
                  family = "gaussian", impacts = FALSE,
                  control.family = list(hyper = zero.variance),
                  control.predictor = list(compute = TRUE),
                  control.compute = list(dic = TRUE, cpo = TRUE),
                  #control.inla = list(print.joint.hyper = TRUE),
                  ##tolerance=1e-20, h=1e-6),
                  verbose = TRUE)

  return(list(mlik = res$mlik[1,1], model = res))
}

#density
dq.rho <- function(x, y, sigma = .15, log =TRUE) {
  dnorm(y, mean = x, sd = sigma, log = log)
}
#random
rq.rho <- function(x, sigma = .15) {
  rnorm(1, mean = x, sd = sigma)
}

#Prior for beta#Uniform [-1.5, 1] using eigenvalues
prior.rho <- function(x, log = TRUE) {
  dunif(x, -1.5, 1, log = log)
}

d<-data2

inlamh.res <- INLAMH(d, fit.inla, 0, rq.rho, dq.rho, prior.rho,
```

```
                              n.sim = 80, n.burnin = 20, n.thin = 5,verbose = TRUE)

b.sim <- do.call(rbind, inlamh.res$b.sim)
model.sim <- inlamh.res$model.sim
summary(b.sim)

par(mfrow = c(1,2))
plot(b.sim, type = "l")
plot(density(b.sim))

print(c("Acceptance rate:", mean(inlamh.res$acc.sim)))

print("Summary statistics of rho:")
c(mean(b.sim), sd(b.sim),
  quantile(b.sim, c(0.025, .5, .975)) )

library(INLABMA)
models <- lapply(model.sim, function(X){X$model})
n.sim <- length(models)
ws <- rep(1/n.sim, n.sim)

listmarg <- c("marginals.fixed", "marginals.hyperpar")
margeff <- mclapply(listmarg, function(X) {
  INLABMA:::fitmargBMA2(models, ws, X)
})

do.call(rbind, lapply(margeff[[1]], inla.zmarginal))
do.call(rbind, lapply(margeff[[2]], inla.zmarginal))

dev.new()
plot(margeff[[1]][[1]], type = "l", main = expression(Urbanization:beta[1]), xlab = "")
dev.new()
plot(margeff[[1]][[2]], type = "l", main = expression(Income:beta[2]), xlab = "")
dev.new()
plot(margeff[[1]][[3]], type = "l", main = expression(Consumption:beta[3]), xlab = "")
dev.new()
plot(margeff[[1]][[4]], type = "l", main = expression(RealEstate:beta[4]), xlab = "")
dev.new()
plot(margeff[[1]][[5]], type = "l", main = expression(ExternalEconomy:beta[5]), xlab = "")
dev.new()
plot(margeff[[1]][[6]], type = "l", main = expression(Technology:beta[6]), xlab = "")
dev.new()
plot(margeff[[1]][[7]], type = "l", main = expression(PopulationAging:beta[7]), xlab = "")
```

2.R code for analysis of China's economic development under the Varying Coefficient Spatial Lag Model (Section 3.3 &3.4 including Section 3.1)

```
library(INLA)
library(spdep)
library(INLABMA)
library(rgdal)
install.packages("spatialreg")
library(spatialreg)
library(spatstat)
library(sp)
library(maptools)
library(parallel)
library(MASS)
library(splines)
library(compiler)
library(spdep)
library(sf)

data_China.economy <- data.frame(read.csv("C:/Users/teng/Desktop/paper/finaldata.csv", head = T,
sep = ","))

library(ggplot2)
library(maptools)
#China.gen<-readShapePoly("Export_Output.shp")
China.gen<-st_read("C:/Users/teng/Desktop/paper/Export_Output.shp")
China.gen$NAME

China_nb <- spdep::poly2nb(China.gen,queen=T)
censored1 <- China.gen$NAME== "台湾"
Chinadata1 <- China.gen[!censored1,]
censored2 <- Chinadata1$NAME== "香港"
Chinadata2 <- Chinadata1[!censored2,]
censored3 <- Chinadata2$NAME== "澳门"
Chinadata3 <- Chinadata2[!censored3,]
total1<-rbind(Chinadata3,Chinadata3)
total2<-rbind(total1,total1)
total3<-rbind(Chinadata3,total2)
total4<-rbind(Chinadata3,total3)
China_nb <- spdep::poly2nb(total4,queen=T)
China_lw <- spdep::nb2listw(China_nb, style="W")
China_W <- as(as_dgRMatrix_listw(China_lw), "CsparseMatrix")

N=186
```

```
data=data_China.economy
x1<-scale(data$x1)
x2<-scale(data$x2)
x3<-scale(data$x3)
x4<-data$x4
x5<-data$x5
x6<-data$x6
U<-scale(data$u)
y<-scale(data$y)

#beta1_(U)
#d=3,r=21,h=d+r+1
h<-20
B.tmp1<- bs(U, df = h,degree = 3, intercept = FALSE)
Bmat1<-as(matrix(B.tmp1,N,h),'sparseMatrix')

#beta2_(U)
#d=3,r=11,h=d+r+1
h<-20
B.tmp2<- bs(U, df = h,degree = 3, intercept = FALSE)
Bmat2<-as(matrix(B.tmp2,N,h),'sparseMatrix')

#beta3_(U)
#d=3,r=11,h=d+r+1
h<-20
B.tmp3<- bs(U, df = h,degree = 3, intercept = FALSE)
Bmat3<-as(matrix(B.tmp3,N,h),'sparseMatrix')

#beta4_(U)
#d=3,r=11,h=d+r+1
h<-20
B.tmp4<- bs(U, df = h,degree = 3, intercept = FALSE)
Bmat4<-as(matrix(B.tmp4,N,h),'sparseMatrix')

#beta5_(U)
#d=3,r=11,h=d+r+1
h<-20
B.tmp5<- bs(U, df = h,degree = 3, intercept = FALSE)
Bmat5<-as(matrix(B.tmp5,N,h),'sparseMatrix')

#beta6_(U)
#d=3,r=11,h=d+r+1
h<-20
B.tmp6<- bs(U, df = h,degree = 3, intercept = FALSE)
```

```
Bmat6<-as(matrix(B.tmp6,N,h),'sparseMatrix')

data=data.frame(x1=x1,x2=x2,x3=x3,x4=x4,x5=x5,x6=x6,y=y,idx=1:N,B.tmp1=B.tmp1,B.tmp2=
B.tmp2,B.tmp3=B.tmp3,B.tmp4=B.tmp4,B.tmp5=B.tmp5,B.tmp6=B.tmp6)
z11<-data$x1*data$B.tmp1.1
z12<-data$x1*data$B.tmp1.2
z13<-data$x1*data$B.tmp1.3
z14<-data$x1*data$B.tmp1.4
z15<-data$x1*data$B.tmp1.5
z16<-data$x1*data$B.tmp1.6
z17<-data$x1*data$B.tmp1.7
z18<-data$x1*data$B.tmp1.8
z19<-data$x1*data$B.tmp1.9
z110<-data$x1*data$B.tmp1.10
z111<-data$x1*data$B.tmp1.11
z112<-data$x1*data$B.tmp1.12
z113<-data$x1*data$B.tmp1.13
z114<-data$x1*data$B.tmp1.14
z115<-data$x1*data$B.tmp1.15
z116<-data$x1*data$B.tmp1.16
z117<-data$x1*data$B.tmp1.17
z118<-data$x1*data$B.tmp1.18
z119<-data$x1*data$B.tmp1.19
z120<-data$x1*data$B.tmp1.20
z21<-data$x2*data$B.tmp2.1
z22<-data$x2*data$B.tmp2.2
z23<-data$x2*data$B.tmp2.3
z24<-data$x2*data$B.tmp2.4
z25<-data$x2*data$B.tmp2.5
z26<-data$x2*data$B.tmp2.6
z27<-data$x2*data$B.tmp2.7
z28<-data$x2*data$B.tmp2.8
z29<-data$x2*data$B.tmp2.9
z210<-data$x2*data$B.tmp2.10
z211<-data$x2*data$B.tmp2.11
z212<-data$x2*data$B.tmp2.12
z213<-data$x2*data$B.tmp2.13
z214<-data$x2*data$B.tmp2.14
z215<-data$x2*data$B.tmp2.15
z216<-data$x2*data$B.tmp2.16
z217<-data$x2*data$B.tmp2.17
z218<-data$x2*data$B.tmp2.18
z219<-data$x2*data$B.tmp2.19
z220<-data$x2*data$B.tmp2.20
```

```
z31<-data$x3*data$B.tmp3.1
z32<-data$x3*data$B.tmp3.2
z33<-data$x3*data$B.tmp3.3
z34<-data$x3*data$B.tmp3.4
z35<-data$x3*data$B.tmp3.5
z36<-data$x3*data$B.tmp3.6
z37<-data$x3*data$B.tmp3.7
z38<-data$x3*data$B.tmp3.8
z39<-data$x3*data$B.tmp3.9
z310<-data$x3*data$B.tmp3.10
z311<-data$x3*data$B.tmp3.11
z312<-data$x3*data$B.tmp3.12
z313<-data$x3*data$B.tmp3.13
z314<-data$x3*data$B.tmp3.14
z315<-data$x3*data$B.tmp3.15
z316<-data$x3*data$B.tmp3.16
z317<-data$x3*data$B.tmp3.17
z318<-data$x3*data$B.tmp3.18
z319<-data$x3*data$B.tmp3.19
z320<-data$x3*data$B.tmp3.20
z41<-data$x4*data$B.tmp4.1
z42<-data$x4*data$B.tmp4.2
z43<-data$x4*data$B.tmp4.3
z44<-data$x4*data$B.tmp4.4
z45<-data$x4*data$B.tmp4.5
z46<-data$x4*data$B.tmp4.6
z47<-data$x4*data$B.tmp4.7
z48<-data$x4*data$B.tmp4.8
z49<-data$x4*data$B.tmp4.9
z410<-data$x4*data$B.tmp4.10
z411<-data$x4*data$B.tmp4.11
z412<-data$x4*data$B.tmp4.12
z413<-data$x4*data$B.tmp4.13
z414<-data$x4*data$B.tmp4.14
z415<-data$x4*data$B.tmp4.15
z416<-data$x4*data$B.tmp4.16
z417<-data$x4*data$B.tmp4.17
z418<-data$x4*data$B.tmp4.18
z419<-data$x4*data$B.tmp4.19
z420<-data$x4*data$B.tmp4.20
z51<-data$x5*data$B.tmp5.1
z52<-data$x5*data$B.tmp5.2
z53<-data$x5*data$B.tmp5.3
z54<-data$x5*data$B.tmp5.4
```

```
z55<-data$x5*data$B.tmp5.5
z56<-data$x5*data$B.tmp5.6
z57<-data$x5*data$B.tmp5.7
z58<-data$x5*data$B.tmp5.8
z59<-data$x5*data$B.tmp5.9
z510<-data$x5*data$B.tmp5.10
z511<-data$x5*data$B.tmp5.11
z512<-data$x5*data$B.tmp5.12
z513<-data$x5*data$B.tmp5.13
z514<-data$x5*data$B.tmp5.14
z515<-data$x5*data$B.tmp5.15
z516<-data$x5*data$B.tmp5.16
z517<-data$x5*data$B.tmp5.17
z518<-data$x5*data$B.tmp5.18
z519<-data$x5*data$B.tmp5.19
z520<-data$x5*data$B.tmp5.20
z61<-data$x6*data$B.tmp6.1
z62<-data$x6*data$B.tmp6.2
z63<-data$x6*data$B.tmp6.3
z64<-data$x6*data$B.tmp6.4
z65<-data$x6*data$B.tmp6.5
z66<-data$x6*data$B.tmp6.6
z67<-data$x6*data$B.tmp6.7
z68<-data$x6*data$B.tmp6.8
z69<-data$x6*data$B.tmp6.9
z610<-data$x6*data$B.tmp6.10
z611<-data$x6*data$B.tmp6.11
z612<-data$x6*data$B.tmp6.12
z613<-data$x6*data$B.tmp6.13
z614<-data$x6*data$B.tmp6.14
z615<-data$x6*data$B.tmp6.15
z616<-data$x6*data$B.tmp6.16
z617<-data$x6*data$B.tmp6.17
z618<-data$x6*data$B.tmp6.18
z619<-data$x6*data$B.tmp6.19
z620<-data$x6*data$B.tmp6.20

z<-cbind(z11,z12,z13,z14,z15,z16,z17,z18,z19,
         z110,z111,z112,z113,z114,z115,
         z116,z117,z118,z119,z120,
         z21,z22,z23,z24,z25,z26,z27,z28,z29,
         z210,z211,z212,z213,z214,z215,
         z216,z217,z218,z219,z220,
         z31,z32,z33,z34,z35,z36,z37,z38,z39,
```

```
          z310,z311,z312,z313,z314,z315,
          z316,z317,z318,z319,z320,
          z41,z42,z43,z44,z45,z46,z47,z48,z49,
          z410,z411,z412,z413,z414,z415,
          z416,z417,z418,z419,z420,
          z51,z52,z53,z54,z55,z56,z57,z58,z59,
          z510,z511,z512,z513,z514,z515,
          z516,z517,z518,z519,z520,
          z61,z62,z63,z64,z65,z66,z67,z68,z69,
          z610,z611,z612,z613,z614,z615,
          z616,z617,z618,z619,z620)
data2=data.frame(y=y,
              z11=z11,z12=z12,z13=z13,z14=z14,z15=z15,z16=z16,z17=z17,z18=z18,z19=z19,
                  z110=z110,z111=z111,z112=z112,z113=z113,z114=z114,z115=z115,
                  z116=z116,z117=z117,z118=z118,z119=z119,z120=z120,
             z21=z21,z22=z22,z23=z23,z24=z24,z25=z25,z26=z26,z27=z27,z28=z28,z29=z29,
                  z210=z210,z211=z211,z212=z212,z213=z213,z214=z214,z215=z215,
                  z216=z216,z217=z217,z218=z218,z219=z219,z220=z220,
             z31=z31,z32=z32,z33=z33,z34=z34,z35=z35,z36=z36,z37=z37,z38=z38,z39=z39,
                  z310=z310,z311=z311,z312=z312,z313=z313,z314=z314,z315=z315,
                  z316=z316,z317=z317,z318=z318,z319=z319,z320=z320,
             z41=z41,z42=z42,z43=z43,z44=z44,z45=z45,z46=z46,z47=z47,z48=z48,z49=z49,
                  z410=z410,z411=z411,z412=z412,z413=z413,z414=z414,z415=z415,
                  z416=z416,z417=z417,z418=z418,z419=z419,z420=z420,
             z51=z51,z52=z52,z53=z53,z54=z54,z55=z55,z56=z56,z57=z57,z58=z58,z59=z59,
                  z510=z510,z511=z511,z512=z512,z513=z513,z514=z514,z515=z515,
                  z516=z516,z517=z517,z518=z518,z519=z519,z520=z520,
             z61=z61,z62=z62,z63=z63,z64=z64,z65=z65,z66=z66,z67=z67,z68=z68,z69=z69,
                  z610=z610,z611=z611,z612=z612,z613=z613,z614=z614,z615=z615,
                  z616=z616,z617=z617,z618=z618,z619=z619,z620=z620,idx=1:N)

data3=data.frame(y=y,
              z11=z11,z12=z12,z13=z13,z14=z14,z15=z15,z16=z16,z17=z17,z18=z18,z19=z19,
                  z110=z110,z111=z111,z112=z112,z113=z113,z114=z114,z115=z115,
                  z116=z116,z117=z117,z118=z118,z119=z119,z120=z120,
             z21=z21,z22=z22,z23=z23,z24=z24,z25=z25,z26=z26,z27=z27,z28=z28,z29=z29,
                  z210=z210,z211=z211,z212=z212,z213=z213,z214=z214,z215=z215,
                  z216=z216,z217=z217,z218=z218,z219=z219,z220=z220,
             z31=z31,z32=z32,z33=z33,z34=z34,z35=z35,z36=z36,z37=z37,z38=z38,z39=z39,
                  z310=z310,z311=z311,z312=z312,z313=z313,z314=z314,z315=z315,
                  z316=z316,z317=z317,z318=z318,z319=z319,z320=z320,
             z41=z41,z42=z42,z43=z43,z44=z44,z45=z45,z46=z46,z47=z47,z48=z48,z49=z49,
                  z410=z410,z411=z411,z412=z412,z413=z413,z414=z414,z415=z415,
                  z416=z416,z417=z417,z418=z418,z419=z419,z420=z420,
```

```
                    z51=z51,z52=z52,z53=z53,z54=z54,z55=z55,z56=z56,z57=z57,z58=z58,z59=z59,
                          z510=z510,z511=z511,z512=z512,z513=z513,z514=z514,z515=z515,
                          z516=z516,z517=z517,z518=z518,z519=z519,z520=z520,
                    z61=z61,z62=z62,z63=z63,z64=z64,z65=z65,z66=z66,z67=z67,z68=z68,z69=z69,
                          z610=z610,z611=z611,z612=z612,z613=z613,z614=z614,z615=z615,
                          z616=z616,z617=z617,z618=z618,z619=z619,z620=z620,U=1:N,idx=1:N)


form=y~-
1+z11+z12+z13+z14+z15+z16+z17+z18+z19+z110+z111+z112+z113+z114+z115+z116+z117+z
118+z119+z120+z21+z22+z23+z24+z25+z26+z27+z28+z29+z210+z211+z212+z213+z214+z215
+z216+z217+z218+z219+z220+z31+z32+z33+z34+z35+z36+z37+z38+z39+z310+z311+z312+z
313+z314+z315+z316+z317+z318+z319+z320+z41+z42+z43+z44+z45+z46+z47+z48+z49+z41
0+z411+z412+z413+z414+z415+z416+z417+z418+z419+z420+z51+z52+z53+z54+z55+z56+z5
7+z58+z59+z510+z511+z512+z513+z514+z515+z516+z517+z518+z519+z520+z61+z62+z63+z
64+z65+z66+z67+z68+z69+z610+z611+z612+z613+z614+z615+z616+z617+z618+z619+z620+f
(U,model='rw2',constr=FALSE)

f1<-y~-
1+z11+z12+z13+z14+z15+z16+z17+z18+z19+z110+z111+z112+z113+z114+z115+z116+z117+z
118+z119+z120+z21+z22+z23+z24+z25+z26+z27+z28+z29+z210+z211+z212+z213+z214+z215
+z216+z217+z218+z219+z220+z31+z32+z33+z34+z35+z36+z37+z38+z39+z310+z311+z312+z
313+z314+z315+z316+z317+z318+z319+z320+z41+z42+z43+z44+z45+z46+z47+z48+z49+z41
0+z411+z412+z413+z414+z415+z416+z417+z418+z419+z420+z51+z52+z53+z54+z55+z56+z5
7+z58+z59+z510+z511+z512+z513+z514+z515+z516+z517+z518+z519+z520+z61+z62+z63+z
64+z65+z66+z67+z68+z69+z610+z611+z612+z613+z614+z615+z616+z617+z618+z619+z620
mmatrix <- model.matrix(f1, data2)

zero.variance = list(prec=list(initial = 15, fixed=TRUE))

fit.inla <- function(data, rho) {
  #, form = form, W = W,
  #   zero.variance = zero.variance) {
  res <- slm.inla(form,d=data3,W=China_W,rho=rho,mmatrix=mmatrix,family="gaussian",
                  impacts = FALSE,verbose = TRUE,
                  control.compute=list(dic=TRUE, cpo=TRUE),
                  control.family = list(hyper = zero.variance))

  return(list(mlik = res$mlik[1,1], model = res))
}

#density
dq.rho <- function(z, y, sigma = .15, log =TRUE) {
  dnorm(y, mean = z, sd = sigma, log = log)
```

```
}
#random
rq.rho <- function(z, sigma = .15) {
   rnorm(1, mean = z, sd = sigma)
}

#Prior for beta#Uniform [-1.5, 1] using eigenvalues
prior.rho <- function(z, log = TRUE) {
   dunif(z, -1.5, 1, log = log)
}

d=data3

inlamh.res <- INLAMH(d, fit.inla, 0, rq.rho, dq.rho, prior.rho,
                                         n.sim = 80, n.burnin = 20, n.thin = 5,verbose = TRUE)

b.sim <- do.call(rbind, inlamh.res$b.sim)
model.sim <- inlamh.res$model.sim
summary(b.sim)

par(mfrow = c(1,2))
dev.new()
plot(b.sim, type = "l")

dev.new()
plot(density(b.sim))

print(c("Acceptance rate:", mean(inlamh.res$acc.sim)))

print("Summary statistics of rho:")
c(mean(b.sim), sd(b.sim),
   quantile(b.sim, c(0.025, .5, .975)) )

library(INLABMA)
models <- lapply(model.sim, function(Z){Z$model})
n.sim <- length(models)
ws <- rep(1/n.sim, n.sim)

listmarg <- c("marginals.fixed", "marginals.hyperpar")
margeff <- mclapply(listmarg, function(Z) {
   INLABMA:::fitmargBMA2(models, ws, Z)
})

do.call(rbind, lapply(margeff[[1]], inla.zmarginal))
```

```
do.call(rbind, lapply(margeff[[2]], inla.zmarginal))

alpha_1_mean<-c(1.436596,0.6150609,-1.103751,0.8656367,-0.7713787,
                0.6146784,-0.5234035,-0.02012718,-1.294814,-0.2931752,
                -0.3157125,-2.818328,1.936401,-0.6094883,0.5921813,
                -1.511275,1.105643,-2.405045,13.16148,0.8896997)

alpha_2_mean<-c(-5.02617,4.783445,-1.503796,1.639728,0.8995706,
                -1.3618,1.500861,-7.761993,-1.933404,-0.6419602,
                -1.520913,4.379324,-5.632491,1.263771,0.7362225,
                1.925781,-0.9862896,-0.4933634,-6.201719,2.839565)

alpha_3_mean<-c(5.375301,-5.344632,2.187043,-2.199526,-0.2979698,
                1.650677,-0.1332786,5.620741,3.26387,2.471467,
                1.176723,-2.05686,1.349031,1.019922,-1.173624,
                -0.2792705,1.699968,-0.8423365,7.815567,-4.948613)

alpha_4_mean<-c(0.2933343,0.09155911,-0.9026959,0.8795074,-0.3669756,
                0.4322279,-0.5713155,0.8480918,-0.2807388,1.019447,
                -0.0540672,0.0237223,2.136537,-0.4458947,0.3464057,
                2.207075,0.5987566,-1.035982,3.39939,0.2582303)

alpha_5_mean<-c(-5.57432,7.494924,0.3136066,-0.3909994,0.0545491,
                -1.144446,-0.1292441,0.8722223,-4.077912,1.192308,
                -0.4907274,0.1578919,-0.04953917,-0.9690163,0.3660724,
                -2.818474,0.1911648,-0.6904327,0.5541602,-6.975207)

alpha_6_mean<-c(6.788092,-7.477281,2.135562,-0.8247346,1.383567,
                1.152539,0.09671441,0.831505,3.454178,-2.923294,
                1.472694,0.6333411,-0.06938734,0.561441,-0.4014693,
                0.171508,-0.8535467,2.857787,-5.812657,6.76256)

alpha_1_mean<-matrix(alpha_1_mean)
beta1_hat<-Bmat1%*%alpha_1_mean
beta1_hat=as.vector(beta1_hat)
write.csv(beta1_hat,file="C:\\Users\\teng\\Desktop\\paper\\ beta1_hat.csv")

alpha_2_mean<-matrix(alpha_2_mean)
beta2_hat<-Bmat2%*%alpha_2_mean
beta2_hat=as.vector(beta2_hat)
write.csv(beta2_hat,file="C:\\Users\\teng\\Desktop\\paper\\ beta2_hat.csv")

alpha_3_mean<-matrix(alpha_3_mean)
beta3_hat<-Bmat3%*%alpha_3_mean
```

```
beta3_hat=as.vector(beta3_hat)
write.csv(beta3_hat,file="C:\\Users\\teng\\Desktop\\paper\\ beta3_hat.csv")

alpha_4_mean<-matrix(alpha_4_mean)
beta4_hat<-Bmat4%*%alpha_4_mean
beta4_hat=as.vector(beta4_hat)
write.csv(beta4_hat,file="C:\\Users\\teng\\Desktop\\paper\\ beta4_hat.csv")

alpha_5_mean<-matrix(alpha_5_mean)
beta5_hat<-Bmat5%*%alpha_5_mean
beta5_hat=as.vector(beta5_hat)
write.csv(beta5_hat,file="C:\\Users\\teng\\Desktop\\paper\\ beta5_hat.csv")

alpha_6_mean<-matrix(alpha_6_mean)
beta6_hat<-Bmat6%*%alpha_6_mean
beta6_hat=as.vector(beta6_hat)
write.csv(beta6_hat,file="C:\\Users\\teng\\Desktop\\paper\\ beta6_hat.csv")

#beta1(U)-beta6(U)
mean(beta1_hat)
mean(beta2_hat)
mean(beta3_hat)
mean(beta4_hat)
mean(beta5_hat)
mean(beta6_hat)

alpha_1_q0.025<-c(-2.052807,-7.483854,-4.161626,-0.8111275,-2.513906,
                  -1.01384,-2.219146,-3.098122,-7.602476,-3.470039,
                  -4.465877,-4.168914,0.3053888,-1.299559,0.07232282,
                  -4.089762,-1.788283,-9.279466,-9.715314,-52.52119)

alpha_2_q0.025<-c(-13.85525,-15.57955,-15.70579,-4.516826,-4.113274,
                  -6.670288,-4.900667,-22.34075,-19.92533,-6.55364,
                  -4.812092,2.233648,-15.7238,-1.798413,-0.9863471,
                  -6.129493,-7.833575,-13.99617,-40.89057,-50.87874)

alpha_3_q0.025<-c(-5.511507,-17.95654,-10.8319,-7.982498,-3.751964,
                  -3.540717,-6.208102,-6.412521,-21.97585,-4.643439,
                  -2.742754,-4.097224,-8.662724,-2.127752,-2.912726,
                  -9.906296,-6.173254,-19.12277,-19.86928,-23.71075)

alpha_4_q0.025<-c(-3.26301,-5.181858,-3.412133,-0.4352526,-1.595833,
                  -0.7053689,-1.796802,-2.069866,-4.549932,0.1418771,
                  -0.7206628,-0.4411143,0.4295846,-1.099147,-0.09563198,
```

```
                    -3.524869,-1.878076,-5.305864,-7.501074,-7.737884)

alpha_5_q0.025<-c(-24.99796,-13.7069,-8.171147,-3.164972,-1.595952,
                    -3.025207,-2.935949,-7.408338,-8.908288,-0.4900391,
                    -1.689051,-0.6127465,-3.873769,-2.774803,-0.047336,
                    -14.8637,-5.641379,-11.89515,-21.75063,-31.01767)

alpha_6_q0.025<-c(-12.82886,-29.12817,-4.63886,-3.473832,-1.313925,
                    -0.9024811,-2.163487,-6.615459,-3.236534,-5.407025,
                    -0.2780587,-0.2866357,-2.394774,-0.6662025,-1.062466,
                    -7.140508,-6.819187,-8.290036,-41.1247,-39.5082)

alpha_1_q0.975<-c(4.915408,8.64857,1.947904,2.533184,0.9641996,
                    2.234697,1.167602,3.040092,4.98791,2.8707,
                    3.806556,-1.470284,3.559794,0.07809705,1.10806,
                    1.07765,3.963023,4.484314,35.78039,54.03485)

alpha_2_q0.975<-c(3.761678,24.98648,12.68471,7.738238,5.902193,
                    3.911771,7.868185,6.811389,15.97047,5.237459,
                    1.760062,6.518688,4.425694,4.307263,2.462841,
                    9.878125,5.875348,12.88643,28.47438,56.28774)

alpha_3_q0.975<-c(16.18553,7.2702,15.08875,3.581007,3.123116,
                    6.825576,5.90935,17.54989,28.36525,9.552098,
                    5.074419,-0.02971338,11.29805,4.15169,0.5444854,
                    9.347348,9.511181,17.3725,35.20944,13.6912)

alpha_4_q0.975<-c(3.823437,5.347743,1.58935,2.18795,0.8546325,
                    1.568392,0.6495733,3.748252,3.959681,1.894368,
                    0.6086807,0.4877145,3.835075,0.2061402,0.7845597,
                    7.940761,3.049791,3.228527,14.22049,8.225141)

alpha_5_q0.975<-c(13.88911,28.45399,8.798235,2.372904,1.696526,
                    0.7293811,2.662885,9.115701,0.7387189,2.863711,
                    0.7030656,0.9228363,3.74689,0.8381402,0.7761325,
                    9.182155,5.970416,10.49917,22.67794,17.01126)

alpha_6_q0.975<-c(26.17257,14.2054,8.834915,1.810535,4.071948,
                    3.181738,2.349215,8.227257,10.12098,-0.4485576,
                    3.21435,1.549298,2.248273,1.777668,0.2623718,
                    7.401558,5.120624,13.9015,29.40247,52.79199)

alpha_1_q0.025<-matrix(alpha_1_q0.025)
beta1_lower<-Bmat1%*%alpha_1_q0.025
```

```
beta1_lower=as.vector(beta1_lower)
write.csv(beta1_lower,file="C:\\Users\\teng\\Desktop\\paper\\beta1_lower.csv")

alpha_1_q0.975<-matrix(alpha_1_q0.975)
beta1_upper<-Bmat1%*%alpha_1_q0.975
beta1_upper=as.vector(beta1_upper)
write.csv(beta1_upper,file="C:\\Users\\teng\\Desktop\\paper\\beta1_upper.csv")


alpha_2_q0.025<-matrix(alpha_2_q0.025)
beta2_lower<-Bmat2%*%alpha_2_q0.025
beta2_lower=as.vector(beta2_lower)
write.csv(beta2_lower,file="C:\\Users\\teng\\Desktop\\paper\\ beta2_lower.csv")

alpha_2_q0.975<-matrix(alpha_2_q0.975)
beta2_upper<-Bmat2%*%alpha_2_q0.975
beta2_upper=as.vector(beta2_upper)
write.csv(beta2_upper,file="C:\\Users\\teng\\Desktop\\paper\\ beta2_upper.csv")

alpha_3_q0.025<-matrix(alpha_3_q0.025)
beta3_lower<-Bmat3%*%alpha_3_q0.025
beta3_lower=as.vector(beta3_lower)
write.csv(beta3_lower,file="C:\\Users\\teng\\Desktop\\paper\\ beta3_lower.csv")

alpha_3_q0.975<-matrix(alpha_3_q0.975)
beta3_upper<-Bmat3%*%alpha_3_q0.975
beta3_upper=as.vector(beta3_upper)
write.csv(beta3_upper,file="C:\\Users\\teng\\Desktop\\paper\\ beta3_upper.csv")

alpha_4_q0.025<-matrix(alpha_4_q0.025)
beta4_lower<-Bmat4%*%alpha_4_q0.025
beta4_lower=as.vector(beta4_lower)
write.csv(beta4_lower,file="C:\\Users\\teng\\Desktop\\paper\\beta4_lower.csv")

alpha_4_q0.975<-matrix(alpha_4_q0.975)
beta4_upper<-Bmat4%*%alpha_4_q0.975
beta4_upper=as.vector(beta4_upper)
write.csv(beta4_upper,file="C:\\Users\\teng\\Desktop\\paper\\beta4_upper.csv")

alpha_5_q0.025<-matrix(alpha_5_q0.025)
beta5_lower<-Bmat5%*%alpha_5_q0.025
beta5_lower=as.vector(beta5_lower)
write.csv(beta5_lower,file="C:\\Users\\teng\\Desktop\\paper\\ beta5_lower.csv")
```

```
alpha_5_q0.975<-matrix(alpha_5_q0.975)
beta5_upper<-Bmat5%*%alpha_5_q0.975
beta5_upper=as.vector(beta5_upper)
write.csv(beta5_upper,file="C:\\Users\\teng\\Desktop\\paper\\ beta5_upper.csv")

alpha_6_q0.025<-matrix(alpha_6_q0.025)
beta6_lower<-Bmat6%*%alpha_6_q0.025
beta6_lower=as.vector(beta6_lower)
write.csv(beta6_lower,file="C:\\Users\\teng\\Desktop\\paper\\beta6_lower.csv")

alpha_6_q0.975<-matrix(alpha_6_q0.975)
beta6_upper<-Bmat6%*%alpha_6_q0.975
beta6_upper=as.vector(beta6_upper)
write.csv(beta6_upper,file="C:\\Users\\teng\\Desktop\\paper\\ beta6_upper.csv")

mean(beta1_lower)
mean(beta1_upper)
mean(beta2_lower)
mean(beta2_upper)
mean(beta3_lower)
mean(beta3_upper)
mean(beta4_lower)
mean(beta4_upper)
mean(beta5_lower)
mean(beta5_upper)
mean(beta6_lower)
mean(beta6_upper)

library(maptools)
library(ggplot2)
beta1_hat=as.vector(beta1_hat)
beta1U<-data.frame(beta1_hat=beta1_hat,x=U)
p <- ggplot(beta1U, aes(x = U))
p1<-p+geom_line(aes(y=
beta1_hat),size=0.7)+xlab('U')+ylab('beta1(U)')+ggtitle('Urbanization:beta1(U)')+
  theme(plot.title = element_text(hjust = 0.5))

beta2_hat=as.vector(beta2_hat)
beta2U<-data.frame(beta2_hat=beta2_hat,x=U)
p <- ggplot(beta2U, aes(x = U))
p<-p+geom_line(aes(y=
beta2_hat),size=0.7)+xlab('U')+ylab('beta2(U)')+ggtitle('Income:beta2(U)')+
  theme(plot.title = element_text(hjust = 0.5))
```

```
beta3_hat=as.vector(beta3_hat)
beta3U<-data.frame(beta3_hat=beta3_hat,x=U)
p <- ggplot(beta3U, aes(x = U))
p<-p+geom_line(aes(y=
beta3_hat),size=0.7)+xlab('U')+ylab('beta3(U)')+ggtitle('Consumption:beta3(U)')+
  theme(plot.title = element_text(hjust = 0.5))

beta4_hat=as.vector(beta4_hat)
beta4U<-data.frame(beta4_hat=beta4_hat,x=U)
p <- ggplot(beta4U, aes(x = U))
p<-p+geom_line(aes(y=beta4_hat),size=0.7)+xlab('U')+ylab('beta4(U)')+ggtitle('Real
Estate:beta4(U)')+
  theme(plot.title = element_text(hjust = 0.5))

beta5_hat=as.vector(beta5_hat)
beta5U<-data.frame(beta5_hat=beta5_hat,x=U)
p <- ggplot(beta5U, aes(x = U))
p<-p+geom_line(aes(y=beta5_hat),size=0.7)+xlab('U')+ylab('beta5(U)')+ggtitle('External
Economy:beta5(U)')+
  theme(plot.title = element_text(hjust = 0.5))

beta6_hat=as.vector(beta6_hat)
beta6U<-data.frame(beta6_hat=beta6_hat,x=U)
p <- ggplot(beta6U, aes(x = U))
p<-p+geom_line(aes(y=
beta6_hat),size=0.7)+xlab('U')+ylab('beta6(U)')+ggtitle('Technology:beta6(U)')+
  theme(plot.title = element_text(hjust = 0.5))
```

```
######### Analysis of spatial and temporal characteristics with varying coefficient spatial lag model

library(ggplot2)
library(maptools)
library(spdep)
China.gen<-readShapePoly("Export_Output.shp")

res2<-slm.inla(form,d=data3,W=China_W,rho=0.27459,mmatrix=mmatrix,family="gaussian",
               impacts = FALSE,verbose = TRUE,
               control.compute=list(dic=TRUE, cpo=TRUE),
               control.family = list(hyper = zero.variance))
```

```
space<-res2$marginals.random$idx

#2015
space15<-res2$marginals.random$idx[1:31]
space15_mean<-lapply(space15,function(x) inla.emarginal(exp,x))
space15_meanbu<-
list(space15_mean$index.1,space15_mean$index.2,space15_mean$index.3,space15_mean$index.
4,space15_mean$index.5,space15_mean$index.6,

space15_mean$index.7,space15_mean$index.8,space15_mean$index.9,space15_mean$index.10,s
pace15_mean$index.11,space15_mean$index.12,

space15_mean$index.13,space15_mean$index.14,space15_mean$index.15,space15_mean$index.
16,space15_mean$index.17,space15_mean$index.18,

space15_mean$index.19,space15_mean$index.20,space15_mean$index.21,space15_mean$index.
22,space15_mean$index.23,space15_mean$index.24,

space15_mean$index.25,space15_mean$index.26,space15_mean$index.27,space15_mean$index.
28,space15_mean$index.29,space15_mean$index.30,
                              0,0,0,space15_mean$index.31)
maps.exp.U15<-data.frame(ID=China.gen$NAME,post.mean=unlist(space15_meanbu))
data.china=attr(China.gen,"data")
attr(China.gen,"data")=data.frame(data.china,maps.exp.U15)
trellis.par.set(axis.line=list(col=NA))
spplot(obj=China.gen,zcol="post.mean",main='',col.regions=gray(99:0/99))

#2016
space16<-res2$marginals.random$idx[32:62]
space16_mean<-lapply(space16,function(x) inla.emarginal(exp,x))
space16_meanbu<-
list(space16_mean$index.32,space16_mean$index.33,space16_mean$index.34,space16_mean$ind
ex.35,space16_mean$index.36,space16_mean$index.37,

space16_mean$index.38,space16_mean$index.39,space16_mean$index.40,space16_mean$index.
41,space16_mean$index.42,space16_mean$index.43,

space16_mean$index.44,space16_mean$index.45,space16_mean$index.46,space16_mean$index.
47,space16_mean$index.48,space16_mean$index.49,

space16_mean$index.50,space16_mean$index.51,space16_mean$index.52,space16_mean$index.
53,space16_mean$index.54,space16_mean$index.55,

space16_mean$index.56,space16_mean$index.57,space16_mean$index.58,space16_mean$index.
```

```
59,space16_mean$index.60,space16_mean$index.61,
                                0,0,0,space16_mean$index.62)
maps.exp.U16<-data.frame(ID=China.gen$NAME,post.mean=unlist(space16_meanbu))
data.china=attr(China.gen,"data")
attr(China.gen,"data")=data.frame(data.china,maps.exp.U16)
trellis.par.set(axis.line=list(col=NA))
spplot(obj=China.gen,zcol="post.mean",main='',col.regions=gray(99:0/99))

#2017
space17<-res2$marginals.random$idx[63:93]
space17_mean<-lapply(space17,function(x) inla.emarginal(exp,x))
space17_meanbu<-
list(space17_mean$index.63,space17_mean$index.64,space17_mean$index.65,space17_mean$ind
ex.66,space17_mean$index.67,space17_mean$index.68,

space17_mean$index.69,space17_mean$index.70,space17_mean$index.71,space17_mean$index.
72,space17_mean$index.73,space17_mean$index.74,

space17_mean$index.75,space17_mean$index.76,space17_mean$index.77,space17_mean$index.
78,space17_mean$index.79,space17_mean$index.80,

space17_mean$index.81,space17_mean$index.82,space17_mean$index.83,space17_mean$index.
84,space17_mean$index.85,space17_mean$index.86,

space17_mean$index.87,space17_mean$index.88,space17_mean$index.89,space17_mean$index.
90,space17_mean$index.91,space17_mean$index.92,
                                0,0,0,space17_mean$index.93)
maps.exp.U17<-data.frame(ID=China.gen$NAME,post.mean=unlist(space17_meanbu))
data.china=attr(China.gen,"data")
attr(China.gen,"data")=data.frame(data.china,maps.exp.U17)
trellis.par.set(axis.line=list(col=NA))
spplot(obj=China.gen,zcol="post.mean",main='',col.regions=gray(99:0/99))

#2018
space18<-res2$marginals.random$idx[94:124]
space18_mean<-lapply(space18,function(x) inla.emarginal(exp,x))
space18_meanbu<-
list(space18_mean$index.94,space18_mean$index.95,space18_mean$index.96,space18_mean$ind
ex.97,space18_mean$index.98,space18_mean$index.99,

space18_mean$index.100,space18_mean$index.101,space18_mean$index.102,space18_mean$ind
ex.103,space18_mean$index.104,space18_mean$index.105,

space18_mean$index.106,space18_mean$index.107,space18_mean$index.108,space18_mean$ind
```

```
ex.109,space18_mean$index.110,space18_mean$index.111,

space18_mean$index.112,space18_mean$index.113,space18_mean$index.114,space18_mean$ind
ex.115,space18_mean$index.116,space18_mean$index.117,

space18_mean$index.118,space18_mean$index.119,space18_mean$index.120,space18_mean$ind
ex.121,space18_mean$index.122,space18_mean$index.123,
                              0,0,0,space18_mean$index.124)
maps.exp.U18<-data.frame(ID=China.gen$NAME,post.mean=unlist(space18_meanbu))
data.china=attr(China.gen,"data")
attr(China.gen,"data")=data.frame(data.china,maps.exp.U18)
trellis.par.set(axis.line=list(col=NA))
spplot(obj=China.gen,zcol="post.mean",main='',col.regions=gray(99:0/99))

#2019
space19<-res2$marginals.random$idx[125:155]
space19_mean<-lapply(space19,function(x) inla.emarginal(exp,x))
space19_meanbu<-
list(space19_mean$index.125,space19_mean$index.126,space19_mean$index.127,space19_mean
$index.128,space19_mean$index.129,space19_mean$index.130,

space19_mean$index.131,space19_mean$index.132,space19_mean$index.133,space19_mean$ind
ex.134,space19_mean$index.135,space19_mean$index.136,

space19_mean$index.137,space19_mean$index.138,space19_mean$index.139,space19_mean$ind
ex.140,space19_mean$index.141,space19_mean$index.142,

space19_mean$index.143,space19_mean$index.144,space19_mean$index.145,space19_mean$ind
ex.146,space19_mean$index.147,space19_mean$index.148,

space19_mean$index.149,space19_mean$index.150,space19_mean$index.151,space19_mean$ind
ex.152,space19_mean$index.153,space19_mean$index.154,
                              0,0,0,space19_mean$index.155)
maps.exp.U19<-data.frame(ID=China.gen$NAME,post.mean=unlist(space19_meanbu))
data.china=attr(China.gen,"data")
attr(China.gen,"data")=data.frame(data.china,maps.exp.U19)
trellis.par.set(axis.line=list(col=NA))
spplot(obj=China.gen,zcol="post.mean",main='',col.regions=gray(99:0/99))

#2020
space20<-res2$marginals.random$idx[156:186]
space20_mean<-lapply(space20,function(x) inla.emarginal(exp,x))
space20_meanbu<-
list(space20_mean$index.156,space20_mean$index.157,space20_mean$index.158,space20_mean
```

```
$index.159,space20_mean$index.160,space20_mean$index.161,

space20_mean$index.162,space20_mean$index.163,space20_mean$index.164,space20_mean$ind
ex.165,space20_mean$index.166,space20_mean$index.167,

space20_mean$index.168,space20_mean$index.169,space20_mean$index.170,space20_mean$ind
ex.171,space20_mean$index.172,space20_mean$index.173,

space20_mean$index.174,space20_mean$index.175,space20_mean$index.176,space20_mean$ind
ex.177,space20_mean$index.178,space20_mean$index.179,

space20_mean$index.180,space20_mean$index.181,space20_mean$index.182,space20_mean$ind
ex.183,space20_mean$index.184,space20_mean$index.185,
                                0,0,0,space20_mean$index.186)
maps.exp.U20<-data.frame(ID=China.gen$NAME,post.mean=unlist(space20_meanbu))
data.china=attr(China.gen,"data")
attr(China.gen,"data")=data.frame(data.china,maps.exp.U20)
trellis.par.set(axis.line=list(col=NA))
spplot(obj=China.gen,zcol="post.mean",main='',col.regions=gray(99:0/99))
```
